# Identifying deer antler *uhrf1* proliferation and *s100a10* mineralization genes using comparative RNA-seq

**DOI:** 10.1186/s13287-018-1027-6

**Published:** 2018-10-31

**Authors:** Dai Fei Elmer Ker, Dan Wang, Rashmi Sharma, Bin Zhang, Ben Passarelli, Norma Neff, Chunyi Li, William Maloney, Stephen Quake, Yunzhi Peter Yang

**Affiliations:** 1 Department of Orthopaedic Surgery, Stanford University, 300 Pasteur Drive, Stanford, CA 94305 USA; 2 Department of Stomatology, Tenth People’s Hospital of Tongji University, 301 Yanchang Road, Shanghai, 200072 China; 3 Department of Bioengineering, Stanford University, Shriram Center 443 Via Ortega, Stanford, CA 94305 USA; 4 Scientific Computing Core, Calico Life Sciences LLC, 1170 Veterans Blvd., South San Francisco, CA 94080 USA; 5 State Key Lab for Molecular Biology of Special Economic Animals, 4899 Juye Street, Changchun, 130112 Jilin China; 6 Department of Applied Physics, Stanford University, 348 Via Pueblo Mall, Stanford, CA 94305 USA; 7 Howard Hughes Medical Institute, 4000 Jones Bridge Road, Chevy Chase, MD 20815 USA; 8 Department of Material Science and Engineering, Stanford University, 496 Lomita Mall, Stanford, CA 94305 USA

**Keywords:** Deer antler, Bone regeneration, Comparative RNA-seq, *uhrf1*, *s100a10*

## Abstract

**Background:**

Deer antlers are bony structures that re-grow at very high rates, making them an attractive model for studying rapid bone regeneration.

**Methods:**

To identify the genes that are involved in this fast pace of bone growth, an in vitro RNA-seq model that paralleled the sharp differences in bone growth between deer antlers and humans was established. Subsequently, RNA-seq (> 60 million reads per library) was used to compare transcriptomic profiles. Uniquely expressed deer antler proliferation as well as mineralization genes were identified via a combination of differential gene expression and subtraction analysis. Thereafter, the physiological relevance as well as contributions of these identified genes were determined by immunofluorescence, gene overexpression, and gene knockdown studies.

**Results:**

Cell characterization studies showed that in vitro-cultured deer antler-derived reserve mesenchyme (RM) cells exhibited high osteogenic capabilities and cell surface markers similar to in vivo counterparts. Under identical culture conditions, deer antler RM cells proliferated faster (8.6–11.7-fold increase in cell numbers) and exhibited increased osteogenic differentiation (17.4-fold increase in calcium mineralization) compared to human mesenchymal stem cells (hMSCs), paralleling in vivo conditions. Comparative RNA-seq identified 40 and 91 previously unknown and uniquely expressed fallow deer (FD) proliferation and mineralization genes, respectively, including *uhrf1* and *s100a10*. Immunofluorescence studies showed that *uhrf1* and *s100a10* were expressed in regenerating deer antlers while gene overexpression and gene knockdown studies demonstrated the proliferation contributions of *uhrf1* and mineralization capabilities of *s100a10*.

**Conclusion:**

Using a simple, in vitro comparative RNA-seq approach, novel genes pertinent to fast bony antler regeneration were identified and their proliferative/osteogenic function was verified via gene overexpression, knockdown, and immunostaining. This combinatorial approach may be applicable to discover unique gene contributions between any two organisms for a given phenomenon-of-interest.

**Electronic supplementary material:**

The online version of this article (10.1186/s13287-018-1027-6) contains supplementary material, which is available to authorized users.

## Background

Deer antlers can account for 28% of the skeletal body weight [1, 2] and are the only known example of a mammalian tissue that regenerates rapidly, easily producing 10 kg or more of bone tissue within a relatively short period of 2 to 3 months [1–7]. Although deer antlers and human bone develop via intramembranous and endochondral modes of ossification [3, 4, 6, 7], deer antlers can grow up to 2 cm per day [3], which sharply contrasts with human femur bone, which grows at 2 cm per year during puberty [8]. Thus, if the molecular components that are involved in this process can be elucidated, this knowledge is expected to advance our understanding of the mammalian bone regeneration and holds promise for rapidly generating large bone volumes for skeletal tissue engineering.

Despite previous efforts [3, 4, 6, 7, 9–20], genes involved in fast antler regeneration remain poorly studied, and functional demonstration of their role(s) in proliferation and bone differentiation is lacking. For example, prior transcriptomic studies of deer antler tissues employed mouse microarrays [12], cDNA-amplified fragment length polymorphism (cDNA-AFLP) [15], or RNA-seq [18–20], but efforts to identify and characterize gene contributions to rapid deer antler growth have been hindered by logistical and technical issues. Such limitations include cross-species hybridization [12], sequence variation [21], the overwhelming number of candidates in transcriptomic datasets [12, 18–20], and the presence of complex spatial and temporal variables among tissue samples [12, 15, 18–20]. Indeed, one of the foremost challenges when working with transcriptomics datasets is determining which detected genes play a role in proliferation, skeletal differentiation, or a completely unrelated cellular process. Thus, previous transcriptomic studies to date have been limited to gene expression profiling with little-to-no characterization of gene contribution to antler proliferation or differentiation.

In this study, we hypothesized that an in vitro comparison of fallow deer (FD) and human RNA-seq data could circumvent several of the aforementioned challenges in identifying FD antler proliferation and mineralization genes. This was based on our reasoning that the fast pace of deer antler regeneration must stem, at least in part, from the rapid proliferation and differentiation of skeletal progenitors. Thus, a comparison of differentially expressed genes obtained from FD- or human-derived cells cultured under identical control and treatment group conditions would eliminate complex in vivo spatial and temporal variables while simplifying bioinformatics analysis to identify proliferation and mineralization gene candidates uniquely expressed in deer (Additional file 1: Figure S1; Additional file 2). Human mesenchymal stem cells (hMSCs) were selected to be compared against as they are a clinically promising therapeutic target for cell-based regenerative medicine [22]. In deer, antlers regenerate from skeletal progenitors found in the periosteum of the cranial appendages called pedicles (pedicle periosteum; PP), which, in turn, generate reserve mesenchyme (RM) cells of the growing antler tip that eventually differentiate into chondrocytes and mineralizing bone cells (Additional file 1: Figure S2) [3, 4, 6, 7]. Based on this, skeletal progenitors were collected from fallow deer (FD) facial periosteum (FP; as a non-antler-derived control), PP, and RM tissues to determine a suitable cell type for comparison with hMSCs. Subsequently, RNA-seq was performed, and genes whose expression were unique to FD antler progenitors were identified based on subtraction analysis between FD and human datasets. Lastly, the function and physiological relevance of these identified genes were determined by immunofluorescence, gene overexpression, and gene knockdown studies.

## Results

### Characterization of deer antler-derived cells

**Fig. 1:**
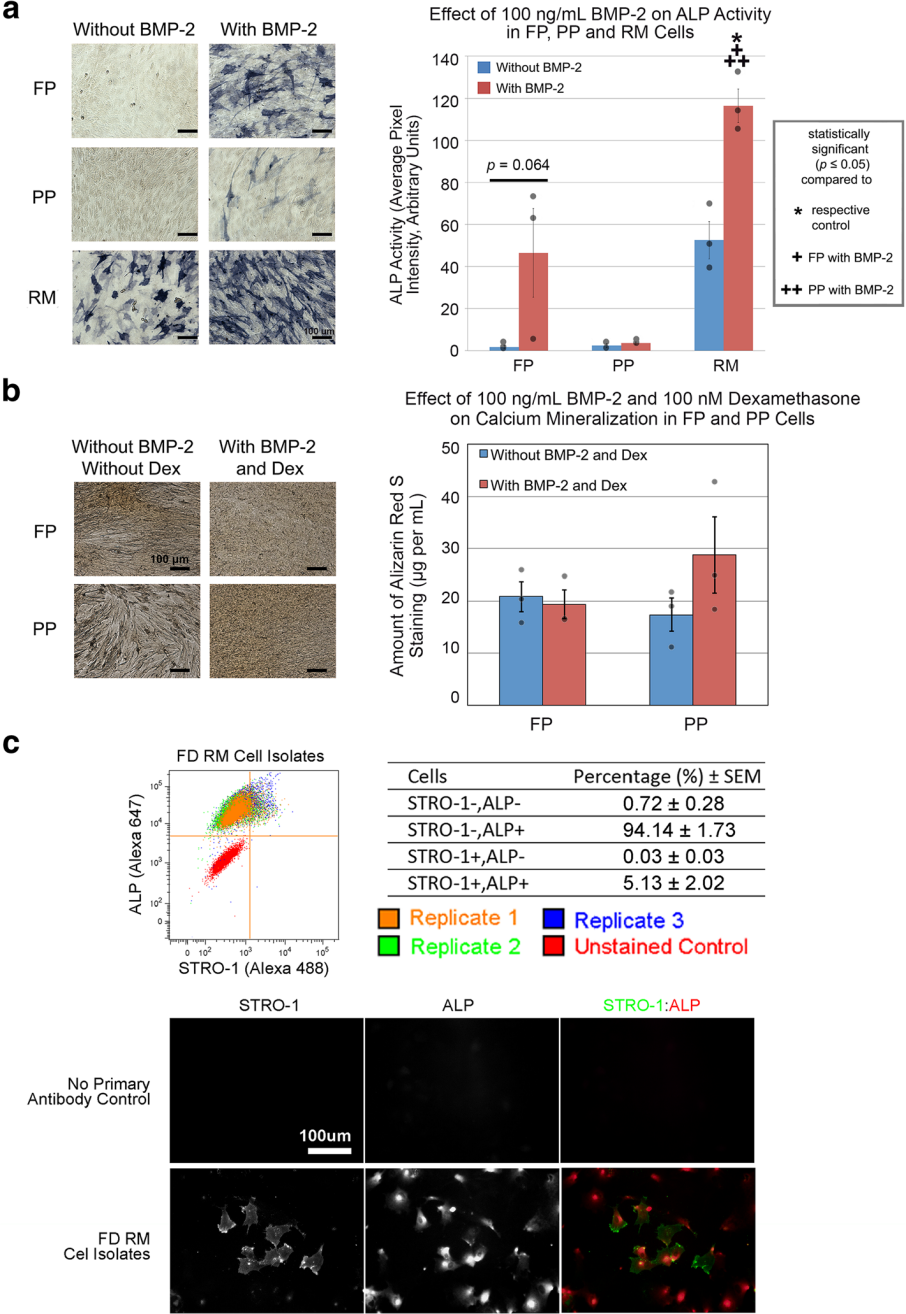
Characterization of in vitro-cultured FD-derived cells. **a** FP and RM cells (isolate 2) cultured with 100 ng/mL BMP-2 for 6 days exhibited increased ALP activity relative to their respective control whereas PP cells (isolate 2) did not. Semi-quantification of ALP activity in FP, PP, and RM cells. **b** FP and PP cells (isolate 2) cultured with 100 ng/mL BMP-2 and 100 nM dexamethasone for 24 days did not exhibit increased Alizarin Red S staining relative to their respective control. Quantification of Alizarin Red S staining in FP and PP cells. **c** FACS analysis of RM cells. The percentage of cells that were negative for STRO1 and ALP, negative for STRO1 but positive for ALP, negative for ALP but positive for STRO1, and positive for both STRO1 and ALP were 0.28–1.25%, 91.83–97.53%, 0.004–0.10%, and 1.20–7.89%, respectively. STRO1 and ALP immunofluorescence staining in RM cells. Green, STRO1-positive cells. Red, ALP-positive cells. Scale bars as indicated. Data were from *n* = 3 isolates (three independent experiments with nine replicates per isolate for ALP and mineralization studies and one independent experiment with three replicates per isolate for FACs studies). Gray circles indicate observed data points. Error bars indicate standard error of mean or SEM. Statistical significance as indicated

In this study, cell characterization studies were performed on FD- and human-derived skeletal progenitors to establish an in vitro model for comparative RNA-seq. Of the cells collected, RM cells exhibited the highest alkaline phosphatase (ALP) activity in the presence of osteogenesis-inducing bone morphogenetic protein-2 (BMP-2; Fig. 1a) while surprisingly, FP and PP cells cultured under mineralization conditions with BMP-2 and dexamethasone for 24 days exhibited little-to-no positive Alizarin Red S staining for calcium deposits (Fig. 1b). Also, fluorescence-activated cell sorting (FACS) analysis and immunofluorescence staining indicated that in vitro-cultured RM cells were similar to their in vivo counterparts [16, 17], with a majority (99.3%) of RM cells expressing ALP and a small subset (5.2%) expressing STRO-1 (Fig. 1c). Together, these studies indicated that in vitro-cultured RM cells were ideal models of deer antler skeletal progenitors for comparison with hMSCs.

### Establishment of an in vitro model that compares deer antler-derived RM cells and hMSCs

**Fig. 2:**
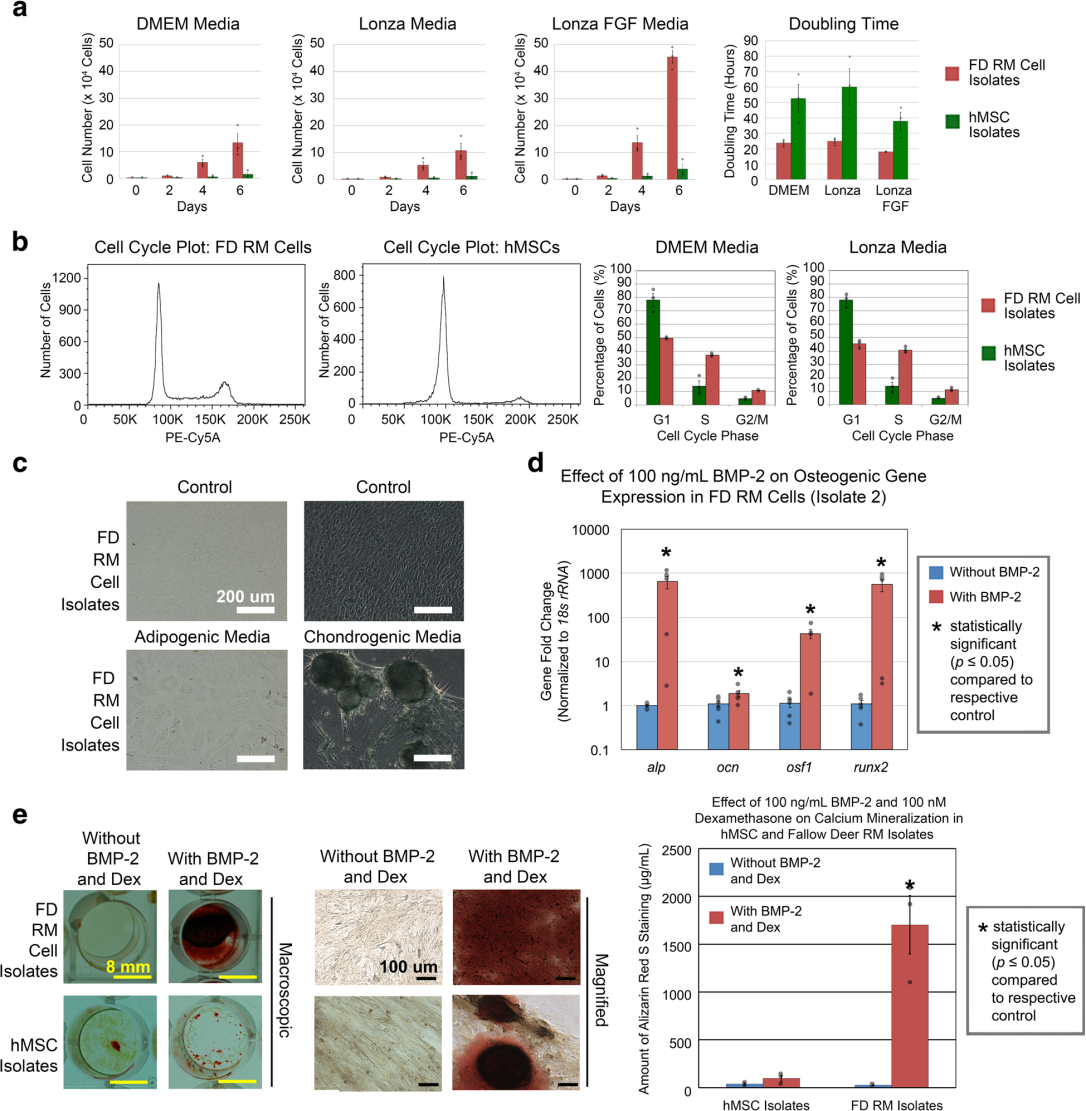
RM cells exhibit increased proliferation and osteogenic differentiation compared to hMSCs. **a** RM cells exhibited increased proliferation relative to hMSCs. **b** Cell cycle analysis showed an increased proportion of RM cells undergoing cell division relative to hMSCs. **c** RM cells were capable of chondrogenic but not adipogenic differentiation. **d** RM cells (isolate 2) cultured with 100 ng/mL BMP-2 for 6 days exhibited increased osteogenic gene expression relative to their respective control. **e** RM cells cultured with 100 ng/mL BMP-2 and 100 nM dexamethasone for 24 days exhibited increased Alizarin Red S staining relative to hMSCs. Scale bars as indicated. Data were from *n* = 3 isolates (three independent experiments with nine replicates per isolate for proliferation and chondrogenic, adipogenic, and mineralization studies and one independent experiment with three replicates per isolate for cell cycle studies) or *n* = 1 isolate (two independent experiments with six replicates for osteogenic gene expression studies). Gray circles indicate observed data points. Error bars indicate SEM. Statistical significance as indicated

Prior to performing RNA-seq, it was necessary to characterize in vitro-cultured RM cells and hMSCs to determine if there is sharply differential osteogenesis in vitro which may reflect the significant bone growth difference in vivo. Successful demonstration of this phenomenon in vitro would thus justify its use for comparative RNA-seq. Proliferation studies involving cells grown in three different mammalian culture media for 6 days demonstrated that RM cells exhibited increased growth relative to hMSCs. RM cells yielded 10.7–45.3 × 10^4^ cells with a doubling time of 17.9–24.7 h whereas hMSCs yielded 1.2–3.9 × 10^4^ cells with a doubling time of 37.8–60.1 h (Fig. 2a; Bartmann et al. [23] and Schallmoser et al. [24]). As such, RM cells yielded 8.6–11.7-fold increase in cell numbers and 2.1–2.4-fold less doubling time compared to hMSCs when controlled for medium formulation (Fig. 2a). Cell cycle analysis showed similar results with a larger percentage of RM cells undergoing cell division relative to hMSCs (Fig. 2b). Differentiation studies demonstrated that RM cells did not undergo adipogenic differentiation but underwent chondrogenesis and osteogenesis (Fig. 2c–e). In osteogenic gene expression studies, RM cells (isolate 2) cultured in the presence for BMP-2 for 6 days showed high upregulation of typical osteogenic genes such as *alkaline phosphatase* (*alp*), *osteocalcin* (*ocn*), *osteoblast-specific factor-1* (*osf-1*), and *runt-related transcription factor-2* (*runx2*) relative to control by 651.1-fold, 1.9-fold, 42.8-fold, and 557.5-fold, respectively (Fig. 2d). In osteogenic mineralization studies, control groups for RM cells and hMSCs (cultured in the absence of BMP-2 and dexamethasone) exhibited 28.0 μg/mL and 39.6 μg/mL Alizarin Red S stain, respectively, whereas treatment groups for RM cells and hMSCs (cultured in the presence of BMP-2 and dexamethasone) exhibited 1702.2 μg/mL and 98.0 μg/mL Alizarin Red S stain, respectively (Fig. 2e). Remarkably, even though identical culture conditions were used, RM cells and hMSCs demonstrated a 62.3-fold and 1.9-fold increase, respectively, in calcium mineralization relative to their respective controls, with RM cells demonstrating 17.4-fold increased levels of calcium mineralization compared to hMSCs despite similar baseline levels in the control groups (Fig. 2e). In addition, RM cells showed 2.0–18.1-fold increase over hMSC-mediated calcium mineralization despite a 5-fold decrease in BMP-2 concentration, as well as 3.1-fold increase in calcium mineralization over hMSC-like C3H10T1/2 cells despite a 1.6-fold longer cell doubling time (Fig. 2; Additional file 1: Figure S3; Ker et al. [25]). Thus, the sharply contrasting proliferation and mineralization capabilities of RM cells and hMSCs justified the use of this in vitro model and were expected to identify genes that orchestrate the differential growth and mineralization rates observed between deer antlers and human bone.

### Identification of deer antler proliferation and mineralization genes using comparative RNA-seq

**Fig. 3:**
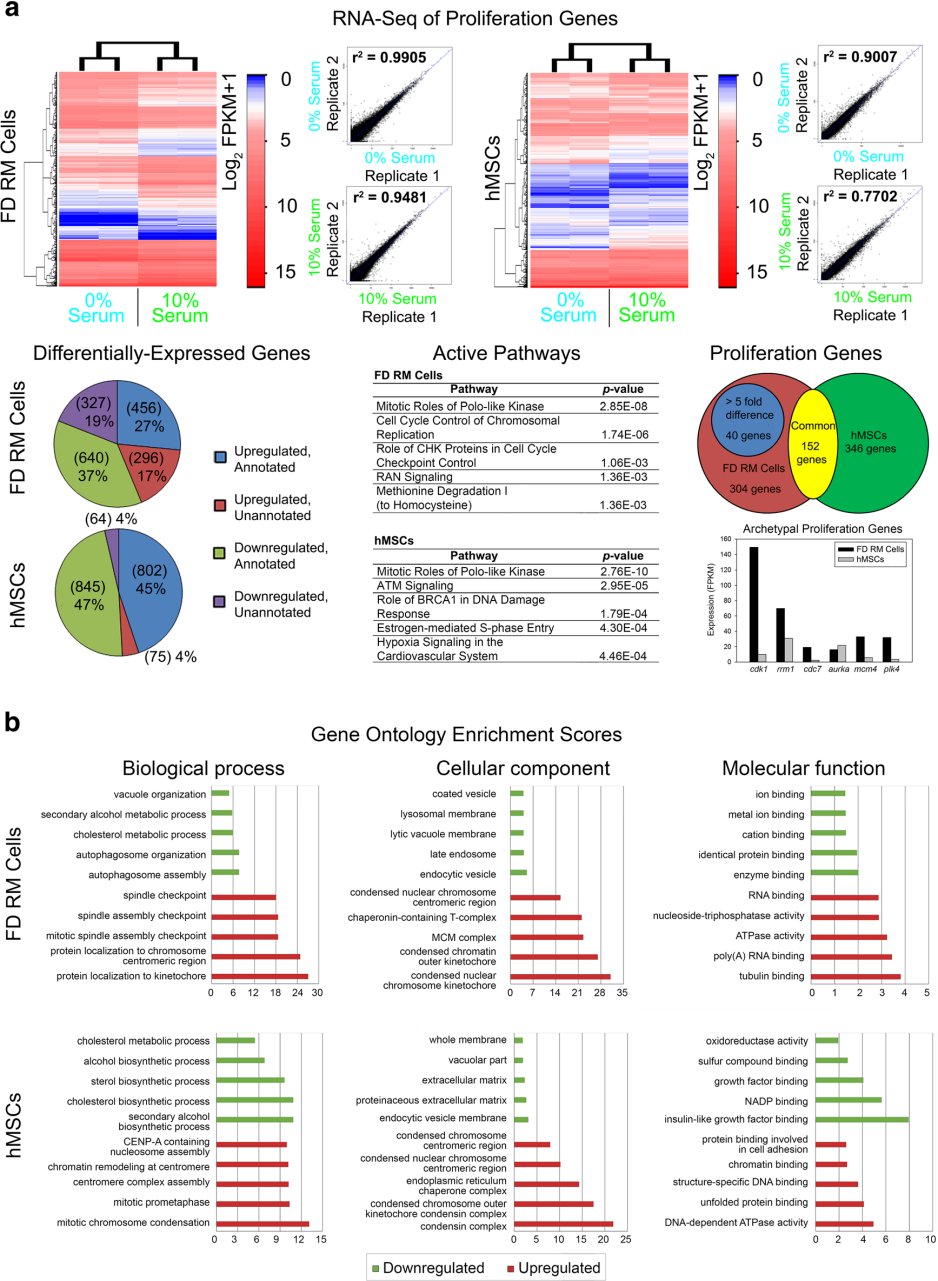
RNA-seq analysis of RM cells and hMSCs under proliferation and mineralization conditions. **a** RNA-seq analysis of RM cells (isolate 2) and hMSCs (isolate 24268) under serum-free (0% serum) and serum-containing (10% serum) conditions identified 40 candidate proliferation genes. Scatterplots indicate the correlation (*r*^2^) between replicates for each condition. FPKM, fragments per kilobase of transcript per million mapped reads. **b** Gene ontology enrichment analysis of RM cells (isolate 2) and hMSCs (isolate 24268) under proliferation conditions. Graphs indicate the top 5 upregulated (red) and downregulated (green) biological processes, cellular components, and molecular functions. Data were from *n* = 1 isolate (one independent experiment with two replicates per group)

**Fig. 4:**
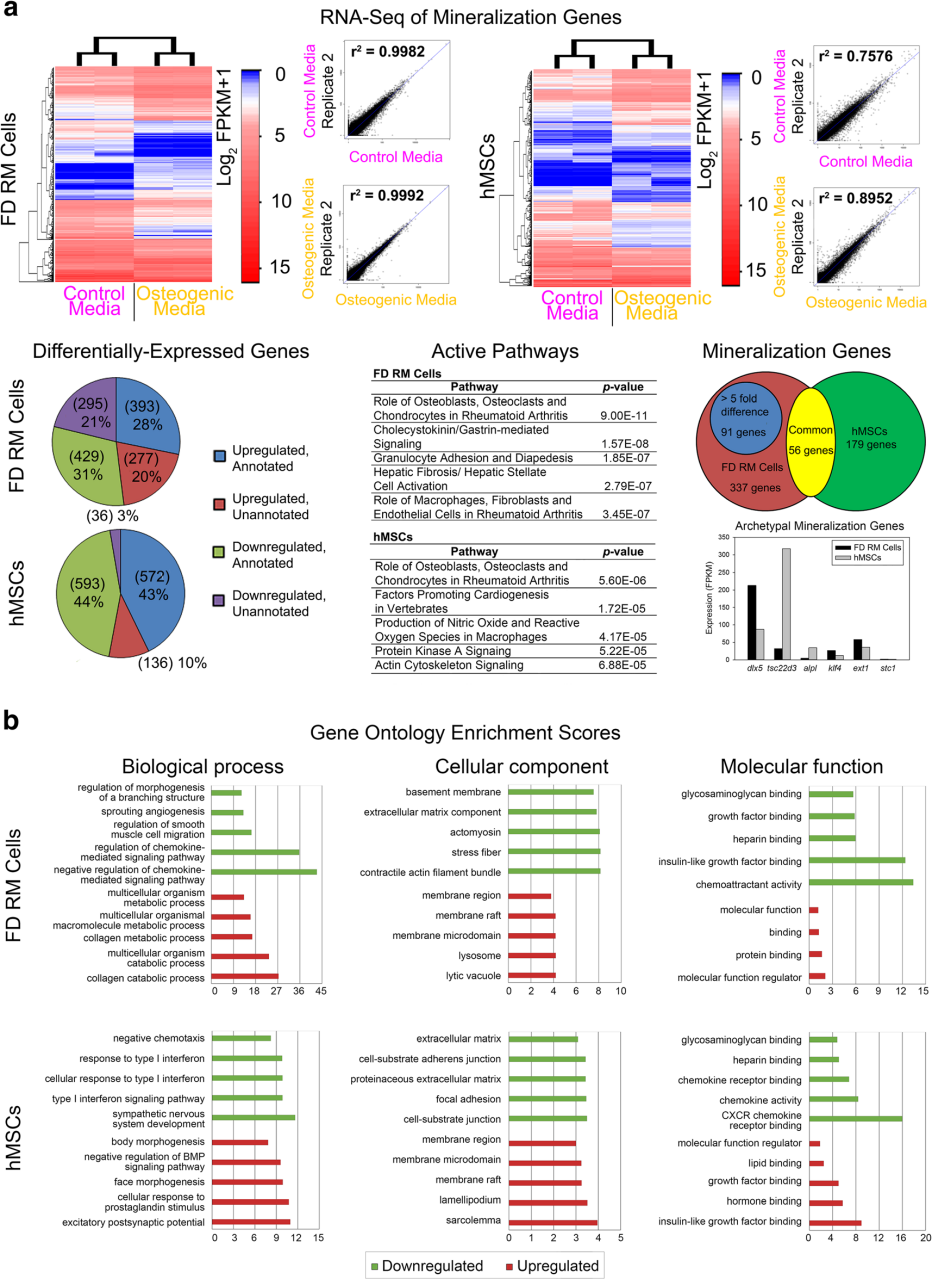
RNA-seq analysis of RM cells and hMSCs under proliferation and mineralization conditions. **a** RNA-seq analysis of RM cells (isolate 2) and hMSCs (isolate 24268) under control (0 ng/mL BMP-2 and 0 nM dexamethasone) and osteogenic (100 ng/mL BMP-2 and 100 nM dexamethasone) conditions identified 91 candidate mineralization genes. Scatterplots indicate the correlation (*r*^2^) between replicates for each condition. FPKM, fragments per kilobase of transcript per million mapped reads. **b** Gene ontology enrichment analysis of RM cells (isolate 2) and hMSCs (isolate 24268) under mineralization conditions. Graphs indicate the top 5 upregulated (red) and downregulated (green) biological processes, cellular components, and molecular functions. Data were from *n* = 1 isolate (one independent experiment with two replicates per group)

To identify the proliferation and mineralization genes that contribute to the fast growth and differentiation observed in our in vitro model, we focused our attention on uniquely expressed genes in RM cells. This was achieved by independently comparing RNA-seq data of RM cells (isolate 2) and hMSCs (isolate 24268) under proliferation (control group, 0% serum; treatment group, 10% serum) as well as mineralization (control group, 0 ng/mL BMP-2 and 0 nM dexamethasone; treatment group, 100 ng/mL BMP-2 and 100 nM dexamethasone) conditions (Figs. 3 and 4). RNA-seq proliferation samples were sequenced to 76,781,962–99,716,096 reads per library with replicates showing a strong correlation of gene expression under serum-free and serum-containing conditions (Fig. 3a and Additional file 1: Table S1). As expected of a non-model organism, a larger percentage of unannotated genes was present in FD (36%) compared to human (8%) RNA-seq data (Fig. 3a). Despite this, Ingenuity Pathway Analysis (IPA) of annotated transcripts showed similar activation of proliferation-associated pathways such as *mitotic roles of polo-like kinase* as well as the expression of typical proliferation genes such as *cdk1*, *rrm1*, *cdc7*, *aurka*, and *plk4* in both datasets (Fig. 3a). Correspondingly, gene ontology analysis showed upregulation of the processes associated with proliferation including mitotic checkpoints and chromosome condensation (Fig. 3b). Also, RNA-seq mineralization samples were sequenced to 62,601,720–86,750,048 reads per library with replicates showing a strong correlation of gene expression under non-mineralization and mineralization conditions (Fig. 4a and Additional file 1: Table S2). Similar to the proliferation dataset, a larger percentage of unannotated genes was present in FD (41%) compared to human (13%) RNA-seq data (Fig. 4a). IPA of annotated transcripts showed similar activation of osteogenic-associated pathways such as *roles of osteoblasts*, *osteoclasts*, and *chondrocytes in rheumatoid arthritis* as well as the expression of typical osteogenic genes such as *dlx5*, *tsc22d3*, *alpl*, *klf4*, *ext1*, and *stc1* in both datasets (Fig. 4a). Correspondingly, gene ontology analysis showed upregulation of processes associated with skeletal catabolism including collagen synthesis as well as face and body morphogenesis (Fig. 4b). Subsequently, subtraction analysis was performed between human and FD datasets for differentially expressed genes. Using the following criteria of highly upregulated (> 5-fold) and uniquely expressed FD genes, 40 proliferation and 91 mineralization candidate genes were identified (Figs. 3a and 4a). Thus, in vitro comparative RNA-seq identified gene candidates that were uniquely expressed in RM cells with a presumed role in rapid deer antler regeneration.

### Validation of in vitro comparative RNA-seq

**Fig. 5:**
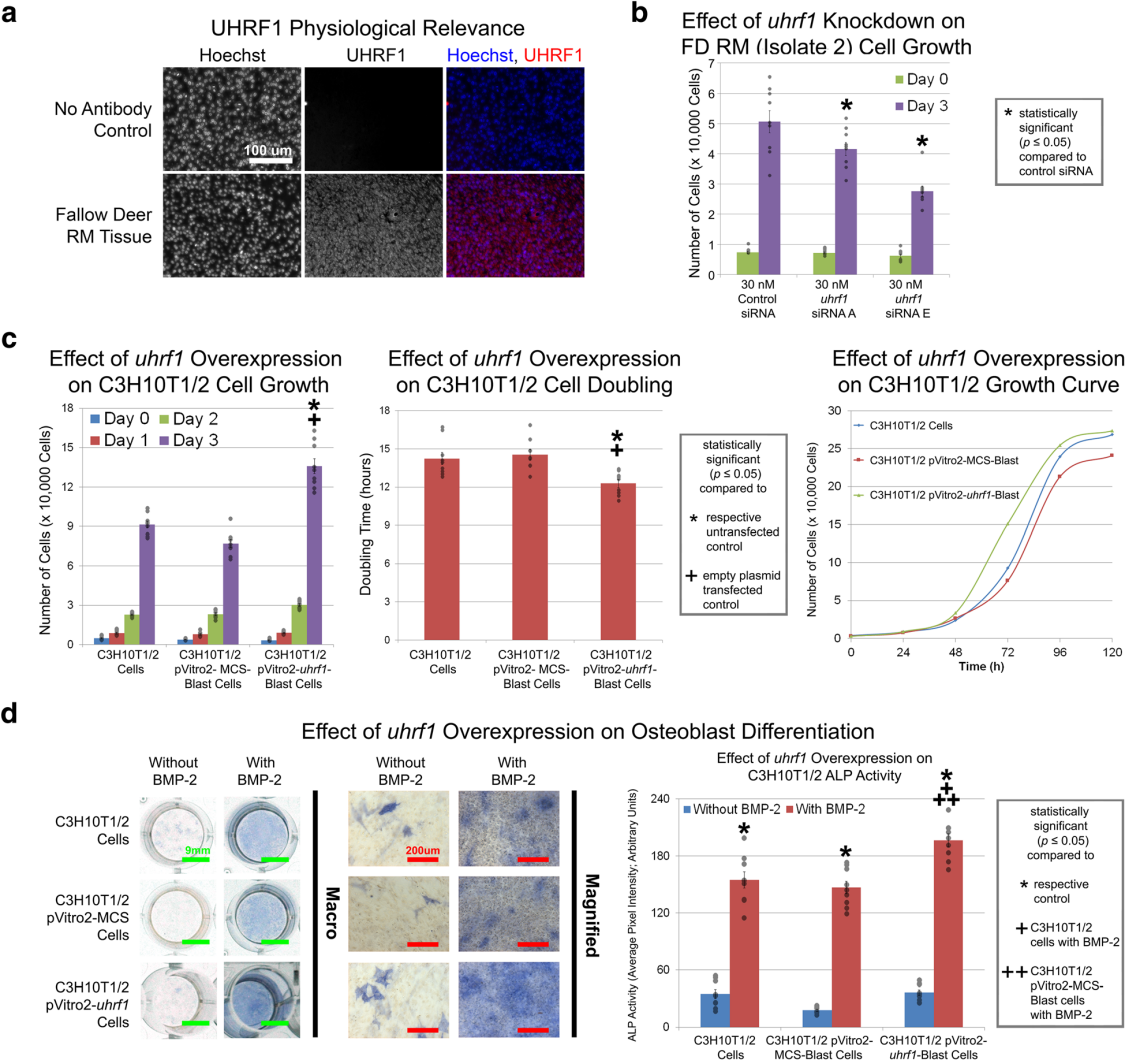
Identification of *uhrf1* as a uniquely expressed proliferation gene using in vitro comparative RNA-seq. **a** UHRF1 immunofluorescence staining in regenerating deer antler tissue. **b** RM cells cultured with 30 nM *uhrf1* siRNAs for 3 days exhibited decreased proliferation relative to mock-transfected control. **c** C3H10T1/2 cells stably transfected with *uhrf1* exhibited increased proliferation relative to untransfected control and empty plasmid control. C3H10T1/2 cells stably transfected with *uhrf1* maintained contact inhibition. Representative growth curves are shown. **d** C3H10T1/2 cells stably transfected with *uhrf1* and cultured with 100 ng/mL BMP-2 for 6 days exhibited increased ALP activity relative to untransfected control and empty plasmid control. Scale bars as indicated. Data were from *n* = 3 isolates (an independent herd for antler immunofluorescence studies) or *n* = 3 independent experiments with nine replicates per group for *uhrf1* knockdown and overexpression proliferation and osteoblast differentiation studies. Gray circles indicate observed data points. Error bars indicate SEM. Statistical significance as indicated

**Fig. 6:**
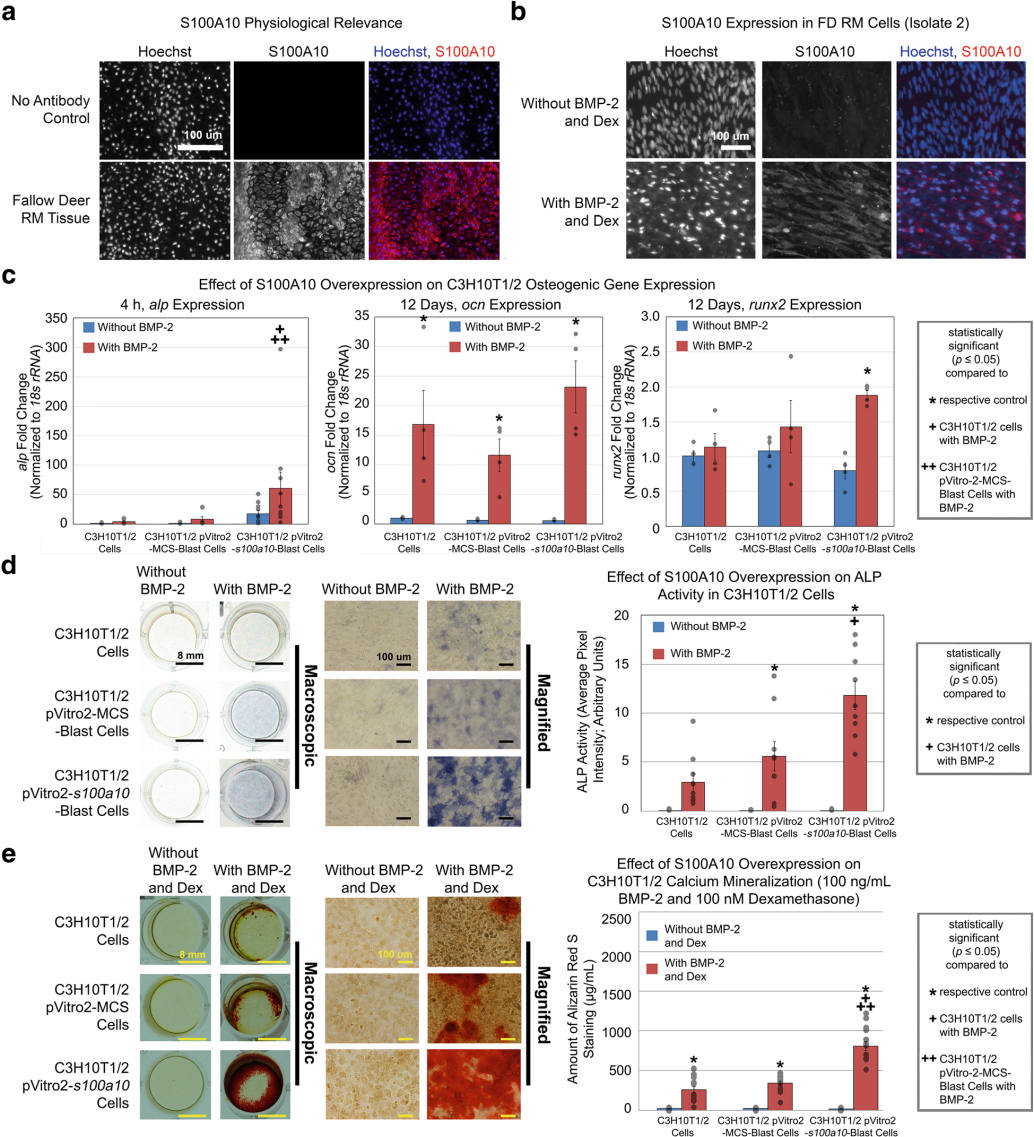
Identification of *s100a10* as a uniquely expressed mineralization gene using in vitro comparative RNA-seq. **a** S100A10 immunofluorescence staining in regenerating deer antler tissue. **b** RM cells (isolate 2) cultured with 100 ng/mL BMP-2 and 100 nM dexamethasone exhibited increased S100A10 expression relative to control. **c** C3H10T1/2 cells stably transfected with *s100a10* and cultured with 100 ng/mL BMP-2 for 4 h exhibited increased *alp* gene expression relative to untransfected control and empty plasmid control. C3H10T1/2 cells stably transfected with *s100a10* and cultured with 100 ng/mL BMP-2 for 12 days exhibited increased *ocn* and *runx2* gene expression relative to their respective control. **d** C3H10T1/2 cells stably transfected with *s100a10* and cultured with 100 ng/mL BMP-2 for 4 days exhibited increased ALP activity relative to untransfected control. **e** C3H10T1/2 cells stably transfected with *s100a10* and cultured in the presence of 100 ng/mL BMP-2 and 100 nM dexamethasone exhibited increased Alizarin Red S staining relative to untransfected control and empty plasmid control. Scale bars as indicated. Data were from *n* = 3 isolates (an independent herd for antler immunofluorescence studies), *n* = 2–3 independent experiments with 4–10 replicates per group for osteogenic gene expression studies, *n* = 3 independent experiments with 9 replicates per group for *s100a10* overexpression ALP studies, and *n* = 5 independent experiments with 15 replicates per group for *s100a10* overexpression mineralization studies. Gray circles indicate observed data points. Error bars indicate SEM. Statistical significance as indicated

To validate the physiological relevance and role of uniquely expressed genes identified by in vitro comparative RNA-seq, a proliferation and mineralization gene candidate was each selected and further assessed in immunofluorescence, gene overexpression, and gene knockdown studies (Figs. 5 and 6). Since chemical- and electroporation-mediated transfections of hMSCs were unsuccessful (data not shown), hMSC-like mouse C3H10T1/2 cells were used in gene overexpression studies.

Of the 40 proliferation gene candidates, FD *uhrf1* was chosen due to its role in epigenetic inheritance [26] and high expression in several cancers [27], suggesting a role for this gene in simultaneously controlling stem cell self-renewal [28] and growth in deer antlers. In immunofluorescence studies, regenerating FD antlers obtained from an independent herd showed UHRF1 expression in RM tissue (Fig. 5a) while supplementation of known mitogens such as fibroblast growth factor-2 (FGF-2) and insulin-like growth factor-1 (IGF-1) in RM cells showed a good correlation between UHRF1 expression and RM cell proliferation (Additional file 1: Figure S4). For example, RM cells exhibited increased UHRF1 expression relative to control when cultured in the presence of IGF-1 under serum-free conditions as well as in the presence of FGF-2 under both serum-free and serum-containing conditions (Additional file 1: Figure S4a and S4b). Correspondingly, increased proliferation was only observed under the aforementioned culture conditions but not in the presence of IGF-1 under serum-containing conditions, where there was no upregulation of UHRF1 (Additional file 1: Figure S4c). Together, these data demonstrate the physiological relevance of *uhrf1* in FD RM cell proliferation and verified the expression pattern of this gene relative to our RNA-seq proliferation data (Fig. 5a and Additional file 1: Figure S4). In gene knockdown studies, RM cells (isolate 2) treated with control siRNA yielded 5.1 × 10^4^ cells, whereas groups treated with siRNAs yielded 2.8–4.2 × 10^4^ cells (Fig. 5b and Additional file 1: Figure S5a). As such, siRNA-mediated knockdown of *uhrf1* inhibited RM cell growth of 17.9–45.5% (Fig. 5b and Additional file 1: Figure S5a). In gene overexpression studies, C3H10T1/2 cells and C3H10T1/2 cells stably transfected with empty plasmid yielded 7.7–9.1 × 10^4^ cells with a doubling time of 13.9–14.3 h while C3H10T1/2 cells stably transfected with *uhrf1* yielded 13.6 × 10^4^ cells with a doubling time of 11.8 h (Fig. 5c). As such, overexpression of FD *uhrf1* in C3H10T1/2 cells (Additional file 1: Figure S5b) increased cell proliferation by 1.49–1.76-fold and decreased cell doubling time by 13.8–15.6% (Fig. 5c) without compromising contact inhibition (Fig. 5c and Additional file 1: Figure S6) or ALP activity (Fig. 5e). This is particularly noteworthy given that untransfected C3H10T1/2 cells already grow rapidly and double every 13.9–15.1 h (Fig. 5c and Ker et al. [25]). Thus, in vitro comparative RNA-seq identified *uhrf1* as contributing towards a previously unknown role in FD antler cell proliferation.

Of the 91 mineralization gene candidates, FD *s100a10*, which has roles in fibrinolysis and intracellular membrane organization [29], was chosen due to the paucity of data regarding its role in osteogenesis, making it as a novel target for further study. In addition, members of the S100 family of proteins such as S100A4 have been reported to be a negative regulator of osteoblast differentiation and matrix mineralization [30]. In immunofluorescence studies, regenerating FD antlers obtained from an independent herd showed S100A10 expression in the cartilage regions of the antler undergoing mineralization (Fig. 6a) while S100A10 was upregulated during osteogenic differentiation of RM cells (Fig. 6b). Together, these data demonstrate the physiological relevance of *s100a10* in FD mineralization and verified the expression pattern of this gene relative to our RNA-seq mineralization data (Fig. 6a, b). In gene overexpression studies, C3H10T1/2 stably transfected with *s100a10* (Additional file 1: Figure S7) showed increased *alp* expression (Fig. 6c) and ALP activity (Fig. 6d) relative to empty plasmid and untransfected controls as well as upregulation of other typical osteogenic genes *ocn* and *runx2* relative to control when cultured in the presence of BMP-2 (Fig. 6c). In addition, C3H10T1/2 stably transfected with *s100a10* exhibited increased calcium mineralization relative to untransfected and empty plasmid controls when in the presence of BMP-2 and dexamethasone (Fig. 6e). Under these culture conditions, C3H10T1/2 cells, C3H10T1/2 cells stably transfected with empty plasmid, and C3H10T1/2 cells stably transfected with *s100a10* exhibited 257.2 μg/mL, 341.9 μg/mL, and 807.9 μg/mL Alizarin Red S stain, respectively (Fig. 6e). As such, overexpression of FD *s100a10* in C3H10T1/2 cells increased cell-mediated mineralization by 2.4–3.1-fold (Fig. 6e). Thus, in vitro comparative RNA-seq identified *s100a10* as contributing towards a previously unknown role in FD antler cell mineralization. Together, these results demonstrated the capability of in vitro comparative RNA-seq analysis to identify uniquely expressed FD proliferation and mineralization genes.

## Discussion

In this study, deer antler-derived RM cells and human bone marrow-derived mesenchymal stem cells were used to establish an in vitro model for comparative RNA-seq (Additional file 1: Figures S1, S2, and S3; Figs. 1 and 2) and identified *uhrf1* and *s100a10* as uniquely expressed deer antler proliferation (Figs. 3 and 5 and Additional file 1: Figures S4, S5, and S6) and mineralization genes (Figs. 4 and 6 and Additional file 1: Figure S7), respectively. The approach developed here may be broadly applied towards studying another biological phenomenon, and the genes identified with this approach will not only advance our understanding of mammalian bone regeneration but also offer promising therapeutic strategies for bone tissue engineering.

Our premise for using an in vitro-based approach was based on several reasons. First, an in vitro approach enabled greater experimental control by allowing culture conditions to define and study the phenomenon-of-interest. Second, the use of identical conditions for culturing deer and human cells not only eliminated complex in vivo spatial and temporal variables but also allowed differential gene expression data from each species to serve as a basis for comparison for identifying uniquely expressed deer antler genes via a simple subtraction analysis. For example, serum-free and serum-containing media conditions were used to identify proliferation genes via differential gene expression. These experiments are independently performed for human and deer cells. Thereafter, subtraction analysis between human and deer proliferation genes would yield uniquely expressed deer proliferation genes. In addition, an in vitro approach reduced the logistical burden for long-term housing of a large, non-model organism such as deer while RNA-seq enabled comprehensive and sensitive detection of transcripts [31] with little bias and error even when non-target species reference genomes are used [32]. Thus, an in vitro approach was expected to identify uniquely expressed deer antler proliferation and mineralization genes.

In establishing this model to identify deer antler proliferation and mineralization genes, hMSCs and RM cells were compared. The basis underlying this choice stems from the significant differences in bone growth between human skeletal and deer antler tissues [3, 4, 6–8] as well as and the therapeutic promise of recapitulating such rapid growth in human skeletal tissues. Although hMSCs and RM cells possess different genetic backgrounds and they do not originate from anatomically equivalent tissues (hMSCs were obtained from the iliac crest whereas RM cells were harvested from the cranial pedicle), a transcriptomic comparison of these cells is still expected to provide important insights as to the genes necessary to stimulate fast proliferation and high mineralization, particularly since hMSCs are a clinically promising therapeutic target for bone tissue engineering [22, 33]. In addition, in vitro-cultured RM cells exhibited high osteogenic capability (Additional file 1: Figure S3, Figs. 1 and 2) and similar cell surface markers (ALP and STRO-1) as their in vivo counterparts [16, 17]. When compared to hMSCs under identical culture conditions, RM cells demonstrated 8.6–11.7-fold increased cell growth and 17.4-fold increased levels of calcium mineralization (Fig. 2), to some extent reflecting the rapid growth and differentiation phenomena observed in regenerating deer antlers. Together, these studies justified the use of in vitro-cultured hMSCs and RM cells to identify deer antler proliferation and mineralization genes.

To identify deer antler proliferation and mineralization genes, RNA-seq was performed. Comparison and subsequent subtraction analysis of transcriptomes under proliferation and mineralization conditions identified 40 proliferation and 91 mineralization genes that were uniquely expressed in RM cells (Fig. 3 and Fig. 4). Further bioinformatics analysis using commercial and public databases showed activation as well as enrichment of proliferation and mineralization pathways or keywords, concurring with the culture conditions employed (Figs. 3 and 4). To validate the contribution of identified genes, *uhrf1* and *s100a10* were chosen for further study based on their potential role in stem cell renewal [28] or novelty, respectively. Although their participation in deer antler biology have not been reported, other studies have indicated that UHRF1 is involved in the proliferation and maturation of colonic T_reg_ cells via epigenetic silencing of CDKNA/P21, an inhibitor of cyclin/cyclin-dependent kinase complexes [34] while related members of the S100 family of proteins such as S100A4 are involved in negative regulation of osteoblast differentiation [30]. Overexpression of FD *uhrf1* in a mouse cell line with hMSC-like characteristics [25] increased cell proliferation without affecting contact inhibition (Fig. 5, Additional file 1: Figures S5 and S6) whereas siRNA-mediated knockdown of *uhrf1* in RM cells decreased cell growth (Fig. 5 and Additional file 1: Figure S5). Similarly, overexpression of FD *s100a10* in this mouse cell line increased osteogenic gene expression, ALP activity, and calcium mineralization (Fig. 6 and Additional file 1: Figure S7). In addition, the physiological relevance of these results was confirmed by immunofluorescence staining of regenerating deer antlers (Figs. 5 and 6). Thus, in vitro comparative RNA-seq identified deer antler proliferation and mineralization genes.

The success of in vitro comparative RNA-seq approach is heavily dependent upon several factors. First, it is necessary to use appropriate in vitro culture conditions that closely model the biological phenomenon-of-interest. In this study, we demonstrated that RM cells rapidly proliferated and exhibited increased calcium mineralization levels relative to hMSCs, which mimicked the phenomenon of rapid bone growth in deer antlers (Fig. 2). However, it is possible that the culture conditions do not accurately reflect growth and differentiation stimuli in regenerating deer antlers, resulting in either false positives or negatives. As such, it is vital to ascertain the physiological relevance of these results by determining gene or protein expression in the relevant biological tissue (Figs. 5 and 6). Despite these limitations, our approach was successful in identifying *uhrf1* and *s100a10* as previously unknown FD antler proliferation and mineralization genes, respectively. However, additional modifications to this study may extend its impact further. These include alternative culture conditions that better mimic physiological conditions such as the use of co-cultures to study antler bone and velvet (skin) paracrine interactions as well as the additional application of more stringent selection criteria such as comparison of in vitro (our current study) and in vivo (deer antler tissue) RNA-seq datasets. Second, it is important to recognize that in vitro comparative RNA-seq performs subtraction analysis between differentially expressed RM genes and its corresponding set of differentially expressed hMSC genes. As such, it is possible that genes which are vital in deer antler proliferation and/or mineralization but are not differentially expressed will not be detected. To address this, our study adopted a strategy of limited biological replication (one isolate, two replicates) with a high number of sequencing reads. This strategy relies on utilizing a large number of sequencing reads to generate increased statistical power for sensitive detection of differential gene expression [35]. Such a strategy would be particularly important for discovering novel, low copy transcripts. Indeed, this approach has been successful in identifying a large number of previously undetected estrogen-related transcripts in breast cancer cells [36]. Third, since the deer genome was only recently sequenced [37], sequencing reads were mapped onto the closely related and well-annotated *Bos taurus* genome. Although such cross-species mapping can result in sequence and expression data loss as well as increased bias and error, these effect sizes are reported to be small within a 100-million-year window and exhibit better mapping performance relative to de novo transcriptome assembly [32]. Despite this, our deer RNA-seq datasets do contain a large percentage of unannotated genes, and this result may be improved by applying orthology-driven Blast mapping.

## Conclusion

In conclusion, we have developed an in vitro model for comparative RNA-seq between FD RM cells and hMSCs to simplify analysis of transcriptomic datasets and for the first time to identify unique genes pertinent to deer antler regeneration. The discovery of these genes advances our understanding of deer antler biology and offer promising strategies for rapid bone regeneration. We envisage a similar comparison strategy can be applied to almost any tissue for identifying the contributions of uniquely expressed genes to a phenomenon of interest.

## Methods

Detailed materials and methods are provided in Additional file 1: Supplementary Information.

### Fallow deer

Tissues were harvested from Lazy Arrow Camatta Ranch (Santa Margarita, CA) and Walking Beam Ranch (Santa Paula, CA) in accordance with approved guidelines established by Stanford University’s Administrative Panel on Laboratory Animal Care (APLAC 28057). Antler tissue was identified [14] and harvested using enzymatic digestion.

### Cell culture

Mouse C3H10T1/2 mesenchymal fibroblasts (American Type Culture Collection; ATCC, Manassas, VA) were maintained in DMEM, 10% FBS, and 1% P/S and used within the first ten passages from the date of receipt. FD cells were maintained in DMEM, 10% FBS and 1% P/S and used between passages 2 and 8. Human mesenchymal stem cells (hMSCs; Lonza, Switzerland) were maintained according to the manufacturer’s instructions and used between passages 4 and 7. Hoechst staining (Anaspec, Fremont, CA) was used to monitor mycoplasma contamination in cell cultures.

### Cell proliferation

Cell proliferation studies were performed in DMEM, 10% FBS, 1% P/S, mesenchymal stem cell growth media (Lonza, Switzerland), and mesenchymal stem cell growth media supplemented with 10 ng/mL fibroblast growth factor-2 (FGF-2; Peprotech, Rocky Hill, NJ). Cells were counted using a Beckman Coulter Z2 Particle Counter (Beckman Coulter, Brea, CA), and cell doubling times were calculated using R-studio (R Studio, Boston, MA, http://www.rstudio.com). Cell cycle studies were performed using propidium iodide/RNAse solution (Cell Signaling Technology, Danvers, MA) on a BD Aria II flow cytometer and analyzed using Flowjo 9.7.5 (Flowjo LLC, Ashland, OR, http://www.flowjo.com).

### Cell differentiation

Adipogenic and chondrogenic differentiation were performed using StemPro Adipogenic (Gibco, Thermo Fisher Scientific, Waltham, MA) and StemPro Chondrogenic Media (Gibco, Thermo Fisher Scientific, Waltham, MA), respectively, according to the manufacturer’s instructions. Osteogenic differentiation was performed using either DMEM, 10% FBS, 1% PS, and 100 ng/mL BMP-2 (Infuse Bone Graft, Medtronic, Sunnyvale, CA) or DMEM, 10% FBS, 1% P/S, 50 μg/mL ascorbic acid, 10 mM β-glycerophosphate, 100 ng/mL BMP-2, and 100 nM dexamethasone (Sigma Aldrich, St. Louis, MO). Osteogenic gene expression was performed on cDNA templates (RNA isolation: Qiagen RNeasy Plus Mini kit, Qiagen, Germany; Reverse transcription: Omniscript kit, Qiagen, Germany) for 40 cycles using TaqMan Gene Expression Mastermix (4369016, Applied Biosystems, Thermo Fisher Scientific, Waltham, MA) on an Applied Biosystems HT7200 thermocycler (Applied Biosystems, Thermo Fisher Scientific, Waltham, MA). Gene expression data were analyzed using SDS 2.2.2 (Applied Biosystems, Thermo Fisher Scientific, Waltham, MA, http://www.thermofisher.com/). ALP activity (Kit 86C, Sigma Aldrich, St. Louis, MO) was detected according to the manufacturer’s instructions. Where necessary, the average pixel intensity was determined using the image histogram tool in Adobe Photoshop as previously described [38, 39]. Osteogenic mineralization was determined using 2% Alizarin Red S stain (Electron Microscopy Sciences, Hatfield, PA) on solvent-extracted samples. Absorbance values from the samples and standards were read at 405 nm using a Tecan Infinite F50 spectrometer (Tecan Trading AG, Switzerland).

### RNA-seq

Proliferation libraries were obtained from cells cultured under DMEM, 0% FBS, and 1% P/S (0% serum) and DMEM, 10% FBS, 1% P/S (10% serum) while mineralization libraries were obtained from cells cultured under DMEM, 10% FBS, 1% P/S, 50 μg/mL ascorbic acid, and 10 mM β-glycerophosphate (control media; without BMP-2 and dexamethasone) and DMEM, 10% FBS, 1% P/S, 50 μg/mL ascorbic acid, 10 mM β-glycerophosphate, 100 ng/mL BMP-2, and 100 nM dexamethasone. RNA was isolated (Qiagen RNeasy Plus Mini kit, Qiagen, Germany), reverse-transcribed into cDNA (Ovation RNA-seq System V2 kit, NuGEN, San Carlos, CA), sheared (S2 Focused-ultrasonicator, Covaris, Woburn, MA), end-repaired, dA-tailed, ligated with custom adaptors, and PCR-amplified for RNA-seq library construction (NEBNext DNA Library Prep Master Mix Set for Illumina, New England Biolabs, Ipswich, MA). Samples were sequenced using 100 base-pair, paired-end RNA-seq technology (HiSeq 2000, Illumina, San Diego, CA), and data were analyzed using several bioinformatics software including Spliced Transcripts Alignment to a Reference (STAR; Version 2.3.0, https://code.google.com/p/rna-star/) [40], SAMtools (Version 0.1.19 http://www.htslib.org/), the Cufflinks package (Version 2.1.1.1, https://github.com/cole-trapnell-lab/cufflinks) [41], the Cummerbund package (http://compbio.mit.edu/cummeRbund/), Ingenuity Pathway Analysis (Qiagen, Germany; https://www.qiagenbioinformatics.com/), and Gene Ontology Enrichment Analysis (http://geneontology.org/page/go-enrichment-analysis). Statistical analyses for RNA-seq were performed as previously described [41]. Gene candidates were identified based on differentially expressed genes that exhibited more than fivefold upregulation in control versus treatment conditions as well as genes that were uniquely expressed in the FD RM cell dataset. From this set of candidates, deer *uhrf1* and *s100a10* genes were manually identified as genes-of-interest based on a literature search (Pubmed; http://www.pubmed.com) of their known biological functions and novelty within the context of mammalian bone biology.

### Gene cloning and transfection

Genes were PCR-cloned (Platinum Blue PCR SuperMix, Invitrogen, Thermo Fisher Scientific, Waltham, MA) from deer cDNA libraries using primers designed from the closely related bovine genome prior to subcloning into a pVitro2-MCS-Blast plasmid (InvivoGen, San Diego, CA). C3H10T1/2 cells were transfected with 2–3 μg plasmid(s) containing the gene-of-interest according to the manufacturer’s instructions (Polyplus, France), and stably transfected cells were selected using 3 μg/mL blasticidin (Invitrogen, Thermo Fisher Scientific, Waltham, MA).

### siRNA

Cells were transfected with 30 nM *uhrf1* siRNA A and E (siRNAs were custom-designed by Santa Cruz Biotechnology Inc., Dallas, TX, based on bovine *uhrf1* sequence) according to the manufacturer’s instructions (Polyplus, France) for 72 h.

### Immunofluorescence staining

Cells were fixed in 4% paraformaldehyde (Electron Microscopy Sciences, Hatfield, PA), blocked with 10% donkey serum (Jackson Immunoresearch Laboratories Inc., West Grove, PA), and incubated with primary antibody followed by secondary antibody incubation with appropriate washes in between. Antibodies included 10 μg/mL mouse anti-Stro-1 (MAB1038, R&D Systems Inc., Minneapolis, MN), 4 μg/mL rabbit anti-alkaline phosphatase (Sc-98652, ALP; Santa Cruz Biotechnology Inc., Dallas, TX), 10 μg/mL rabbit anti-UHRF1 (Sc98704, Santa Cruz Biotechnology Inc., Dallas, TX), 1 μg/mL mouse anti-S100A10 (Ab89438, Abcam Inc., Cambridge, MA), 15 μg/mL donkey anti-mouse Alexa 488 (715-545-150, Jackson Immunoresearch Laboratories Inc., West Grove, PA), and 15 μg/mL donkey anti-rabbit Alexa 647 (711-605-152, Jackson Immunoresearch Laboratories Inc., West Grove, PA). Where necessary, antigen retrieval was performed using antigen retrieval buffer solution (IHC World LLC, Woodstock, MD) at 80–90 °C for 30–60 min prior to antibody incubation. Average pixel intensity was determined using the image histogram tool in Adobe Photoshop as previously described [38, 39].

### Statistical analysis

Statistical analyses involving RNA-seq were performed by Cufflinks and R-Studio [41]. Statistical significance for differentially expressed genes was established at *p* ≤ 0.05 and *q* ≤ 0.05. Statistical analyses not involving RNA-seq were performed using IBM SPSS Statistics for Windows 22 (IBM Corp., North Castle, NY, http://www.ibm.com). Quantitative data was presented as means ± standard error of mean (mean ± SEM) where appropriate. Relative fold changes for PCR data were log transformed in order to make the data distribution more symmetrical [42]. The Shapiro-Wilk test and the Levene test were used to determine whether data were normally distributed and contained equal variances among groups, respectively. For two mean comparisons, *p* values were computed via the *t* test. *p* values were calculated using pooled and separate variance for data with equal and unequal variances, respectively. For more than two mean comparisons, *p* values were computed via analysis of variance (ANOVA). If the majority of the data were normally distributed or there was an equal variance among groups, *p* values were calculated using ANOVA followed by Tukey’s honest significant difference *post hoc* multiple comparison test [43, 44]. Otherwise, *p* values were calculated using Welch’s ANOVA followed by Games-Howell *post hoc* multiple comparison test [45]. Statistical significance was established at *p* ≤ 0.05.

## Additional files

Additional file 1: Supplementary figures. (PDF 2760 kb)

Additional file 2: Comparison of Differentially Expressed Genes from Fallow Deer RM and Human Mesenchymal Stem Cells. (XLSX 13100 kb)

## Abbreviations

ALP: Alkaline phosphatase
ANOVA: Analysis of variance
BMP-2: Bone morphogenetic protein-2
cDNA-AFLP: cDNA-amplified fragment length polymorphism
FACS: Fluorescence-activated cell sorting
FD: Fallow deer
FGF-2: Fibroblast growth factor-2
FP: Facial periosteum
hMSCs: Human mesenchymal stem cells
IGF-1: Insulin-like growth factor-1
IPA: Ingenuity Pathway Analysis
*ocn*: *Osteocalcin*
*osf-1*: *Osteoblast-specific factor-1*
PP: Pedicle periosteum
RM: Reserve mesenchyme
*runx2*: *Runt-related transcription factor-2*
STAR: Spliced Transcripts Alignment to a Reference
