# Supplementary material for: Identifying deer antler uhrf1 proliferation and s100a10 mineralization genes using comparative RNA-seq
Source: Stem Cell Res Ther. 2018 Oct 31;9:292. doi: 10.1186/s13287-018-1027-6 (PMC6208050; doi:10.1186/s13287-018-1027-6)
Supplement: Supplementary file 1 — Supplementary figures. (PDF 2760 kb) [file 13287_2018_1027_MOESM1_ESM.pdf]

Identifying deer antler *uhrf1* proliferation and *s100a10* mineralization genes using comparative RNA-seq

D.F.E. Ker[1], D. Wang[2,1], R. Sharma[3], B. Zhang[1], B. Passarelli[4], N.F. Neff[3], C. Li[5], W. Maloney[1], S.R. Quake[3,6,7] and Y.P. Yang[1,3,8*]

[1] Department of Orthopaedic Surgery, Stanford University, 300 Pasteur Drive, Stanford, California, 94305, USA.

[2] Department of Stomatology, Tenth People's Hospital of Tongji University, 301 Yanchang Road, Shanghai, 200072, China.

[3] Department of Bioengineering, Stanford University, Shriram Center 443 Via Ortega, Stanford, California, 94305, USA.

[4] Scientific Computing Core, Calico Life Sciences LLC, 1170 Veterans Blvd., South San Francisco, CA 94080, USA.

[5] State Key Lab for Molecular Biology of Special Economic Animals, 4899 Juye Street, Changchun, Jilin,130000, China.

[6] Department of Applied Physics, Stanford University, 348 Via Pueblo Mall, Stanford, California, 94305, USA.

[7] Howard Hughes Medical Institute, 4000 Jones Bridge Road, Chevy Chase, MD 20815, USA.

[8] Department of Material Science and Engineering, Stanford University, 496 Lomita Mall, Stanford, California, 94305, USA.

Corresponding Author: Yunzhi Peter Yang, Ph.D., Department of Orthopaedic Surgery, Stanford University, 300 Pasteur Drive, Edwards R 155, Stanford, CA, 94305 (ypyang@stanford.edu)

Keywords: Deer antler, bone regeneration, Comparative RNA-seq, *uhrf1*, *s100a10*

This PDF file includes

- Detailed Materials and Methods
- Supplementary Figures S1-S7
- Supplementary Tables S1-S2
- Supplementary References

Other Supplementary Materials for this manuscript includes the following

- Excel File containing Differentially Expressed Human and Deer Genes
- Transcriptome Sequencing Data (Available from https://www.ncbi.nlm.nih.gov/geo/ under accession number GSE89796)

Detailed Materials and Methods

Isolation of Fallow Deer (FD; Dama dama) cells. Various skeletal progenitor cells were isolated from FD bone tissues at a local deer ranch (Lazy Arrow Camatta Ranch, Santa Margarita, CA) in accordance with the guidelines established by Stanford University's Administrative Panel on Laboratory Animal Care. Tissue from facial (mandibular) periosteum (FP), pedicle periosteum (PP) and reserve mesenchyme (RM) were obtained from three FD (2 – 3 years old) during the first few weeks of antler growth when the antlers were approximately 4 – 7 inches in height. A sample size of three fallow deer were chosen to account for isolate-to-isolate variability. To obtain FD FP, a No.21 scalpel (Feather Safety Razor Co. Ltd, Japan) was used to make a 6 cm skin incision directly over the mandible to expose the tissue. Following this, FD FP tissue was identified and harvested. To obtain FD PP, a No. 21 scalpel was used to make a 6 cm skin incision directly over the pedicle to expose the tissue. Following this, FD PP tissue was identified (1) and harvested. To obtain FD RM, a Gigli bone saw (Miltex GmbH, Germany and Medicon eG, Germany) was used to isolate the antler from the pedicle and a No. 21 scalpel was used to bisect the deer antler in a sagittal manner. Following this, FD RM tissue was identified as a layer of tissue beneath the antler dermis (1) and harvested. The harvested tissues were subsequently stored in cell culture media on ice for transport back to the lab. The cell culture media consisted of high glucose Dulbecco's Modified Eagle Medium, (DMEM; Gibco, Thermo Fisher Scientific, Waltham, MA), 10 % fetal bovine serum (FBS; Gibco, Thermo Fisher Scientific, Waltham, MA), 1 % Penicillin-Streptomycin at 10,000 U/mL (P/S; Gibco, Thermo Fisher Scientific, Waltham, MA), and 25 mM HEPES (pH 7.4; Gibco, Thermo Fisher Scientific, Waltham, MA). To isolate FD cells from tissues, samples were first digested in 0.2 % Collagenase/Dispase solution (Roche, Switzerland) at 37 °C, shaking at 175 rpm for 2 – 3 hours followed by 0.125 % trypsin (Gibco, Thermo Fisher Scientific, Waltham, MA) at 37 °C, shaking at 175 rpm for 0.5 – 1 hour. At each enzyme-dissociation step, cells were decanted and placed on ice. Eventually, collagenase/dispase- and trypsin-dissociated cells were pooled and pelleted by centrifugation (500 g at 4 °C for 5 min). The cells were then resuspended in DMEM, 10 % FBS and 1 % P/S and transferred into T75 tissue culture flasks along with any remaining tissue pieces. FD cells were maintained as individual isolates, expanded in culture and used between passages 4 – 10 for subsequent characterization and RNA-seq studies.

Mammalian cell culture. Various skeletal progenitor cells from mouse, human and deer were used. Mouse C3H10T1/2 mesenchymal fibroblasts (American Type Culture Collection; ATCC, Manassas, VA) and FD cells were maintained in DMEM, 10 % FBS and 1 % P/S. Human mesenchymal stem cells (hMSCs; Lonza, Switzerland) were maintained in Mesenchymal Stem Cell Growth Media (MSCGM BulletKit PT-3238 and PT-4105, Lonza, Switzerland) according to the manufacturer's instructions and used between passages 4 – 7. Cells were kept at 5 % CO_2 and 37 °C. Prior to reaching confluency, cells were passaged using 0.05 % trypsin (Gibco, Thermo Fisher Scientific, Waltham, MA). Hoechst staining (Anaspec, Fremont, CA) determined that cell cultures were free of mycoplasma contamination.

Surface characterization of FD RM cells. FD RM cells were subjected to cell surface characterization using fluorescence-activated cell sorting (FACS) analysis and immunofluorescence staining.

For FACS analysis, cells were seeded into T75 tissue culture flasks at a density of 0.13 – 0.27 x 10^4 cells/cm^2 overnight. The following day (Day 0), media were changed to DMEM, 10 % FBS, 1 % P/S. After 2 – 3 days, cells were washed in PBS (Gibco, Thermo Fisher Scientific, Waltham, MA), dissociated with cell stripper (Mediatech, Corning Inc., Corning NY), pelleted by centrifugation (210 g at 4 °C for 5 min) and fixed in 4 % paraformaldehyde (Electron Microscopy Sciences, Hatfield, PA) for 10 min. Following this, antibody staining was performed. Cells were pelleted by centrifugation (210 g at 4 °C for 5 min), re-suspended in 10 % donkey serum (Jackson Immunoresearch Laboratories Inc., West Grove, PA) for 20 min, pelleted by centrifugation (210 g at 4 °C for 5 min) and incubated in 10

µg/mL mouse anti-Stro-1 (MAB1038, R & D Systems Inc., Minneapolis, MN) and 4 µg/mL rabbit anti-Alkaline Phosphatase (Sc-98652, ALP; Santa Cruz Biotechnology Inc., Dallas, TX) primary antibodies overnight at 4 °C. The following day, cells were pelleted by centrifugation (210 g at 4 °C for 5 min), washed in PBS, incubated in 15 µg/mL donkey anti-mouse Alexa 488 (715-545-150, Jackson Immunoresearch Laboratories Inc., West Grove, PA) and 15 µg/mL donkey anti-rabbit Alexa 647 (711-605-152, Jackson Immunoresearch Laboratories Inc., West Grove, PA) secondary antibodies for 30 min, pelleted by centrifugation (210 g at 4 °C for 5 min) and washed in wash buffer (0.1 % Bovine Serum Albumin in PBS; Santa Cruz Biotechnology Inc., Dallas, TX) prior to analysis. Analyses were performed on a BD Aria II flow cytometer (BD Biosciences, San Jose, CA) and data were analyzed using Flowjo 9.7.5 (Flowjo LLC, Ashland, OR, http://www.flowjo.com).

For immunofluorescence staining, cells were seeded into 35 mm glass-bottom dishes at a density of 0.5 x 10^4 cells/cm^2 overnight. The following day (Day 0), media were changed to DMEM, 10 % FBS, 1 % P/S. After 2 days, cells were fixed in 4 % paraformaldehyde. Following this, antibody staining was performed. Cells were incubated in 10 % donkey serum for 20 min and incubated in 10 µg/mL mouse anti-Stro-1 (MAB1038, R&D Systems, Minneapolis, MN) and 4 µg/mL rabbit anti-ALP (Sc-98652, Santa Cruz Biotechnology Inc., Dallas, TX) primary antibody overnight at 4 °C. The following day, cells were washed 3 times in wash buffer (5 min each), incubated in 15 µg/mL donkey anti-mouse Alexa 488 (715-545-150, Jackson Immunoresearch, West Gove, PA) and 15 µg/mL donkey anti-rabbit Alexa 647 (711-605-152, Jackson Immunoresearch, West Gove, PA) secondary antibody for 1 h at 25 °C and washed 5 times in wash buffer (5 min each). Fluorescence images were acquired using an inverted Zeiss AxioObserver Z1 microscope (Zeiss Microimaging, Thornwood, NY) equipped with an X-Cite® Series 120Q metal halide lamp (Zeiss Microimaging, Thornwood, NY) and an Axiocam MRm camera (Zeiss Microimaging, Thornwood, NY).

Characterization of FD RM cells and hMSCs for cell proliferation studies. The proliferative capacity of FD RM cells and hMSCs were determined by cell counting studies and cell cycle analysis.

For cell counting studies, cells were seeded into 48-well plates at a density of 0.26 x 10^4 cells/cm^2 overnight (Day 0). Three different media formulation were used – 1) DMEM, 10 % FBS, 1 % P/S, 2) Mesenchymal Stem Cell Growth Media and 3) Mesenchymal Stem Cell Growth Media supplemented with 10 ng/mL fibroblast growth factor-2 (FGF-2; Peprotech, Rocky Hill, NJ). Media were changed every 48 h. At 2, 4 and 6 days, cells were counted using an automated cell counter (Beckman Coulter Z2 Particle Counter, Beckman Coulter, Brea, CA). Cell doubling times were calculated using R-studio (R Studio, Boston, MA, http://www.rstudio.com) by visually determining the exponential phase of growth, and plotting the log of the cell counts against time to determine the slope (ln(2)/slope = doubling time) for each sample.

For cell cycle analysis, cells were seeded into T75 tissue culture flasks at a density of 0.27 x 10^4 cells/cm^2 overnight. The following day (Day 0), media were changed to DMEM, 10 % FBS, 1 % P/S. After 4 days, cells were dissociated with 0.05 % trypsin, resuspended in PBS, pelleted by centrifugation (210 g at 4 °C for 5 min for FD RM cells and 500 g at 25 °C for 5 min for hMSCs), fixed in 4 % paraformaldehyde for 10 min, pelleted by centrifugation (210 g at 4 °C for 5 min for FD RM cells and 500 g at 25 °C for 5 min for hMSCs) and stored in PBS at 4 °C until analysis. On the day of analysis, cells were pelleted by centrifugation (210 g at 4 °C for 5 min for FD RM cells and 500 g at 25 °C for 5 min for hMSCs), permeabilized with 0.1 % Triton X-100 in PBS (Sigma Aldrich, St. Louis, MO) for 15 min and stained using propidium iodide/RNAse solution (Cell Signaling Technology, Danvers, MA) for 30 min. Analyses were performed on a BD Aria II flow cytometer and data were analyzed using Flowjo 9.7.5. For each cell cycle distribution, data were fitted to a Watson-Pragmatic model.

Characterization of FD cells and hMSCs for cell differentiation studies. The mesenchyme lineage commitment of FD cells and hMSCs were determined by several cell differentiation assays. The ability of FD cells to differentiate into adipocytes and chondrocytes was confirmed by Oil Red O

staining and Alcian Blue staining, respectively. The ability of FD cells to differentiate into osteoblasts was confirmed by osteogenic gene expression, ALP staining and Alizarin Red S staining whereas the ability of hMSCs to differentiate into osteoblasts was confirmed by Alizarin Red S staining.

For adipogenic studies, cells were seeded into 24-well plates at a density of 1.57 x 10^4 cells/cm^2 overnight. The following day (Day 0), media were changed to StemPro Adipogenic Basal Media (Gibco, Thermo Fisher Scientific, Waltham, MA), 10 % FBS, 1 % P/S (Control media) or StemPro Adipogenic Differentiation Kit Media (Gibco, Thermo Fisher Scientific, Waltham, MA; Adipogenic media). Media were changed every 72 h. After 24 days, cells were washed with PBS, fixed with 10 % neutral buffered formalin for 30 min, stained with 0.5 % Oil Red O (in 60 % isopropanol; Electron Microscopy Sciences, Hatfield, PA) for 30 min, washed with 60 % isopropanol and air-dried. Brightfield images were acquired using an inverted Zeiss AxioObserver Z1 microscope equipped with an Axiocam ICC 1 color camera (Zeiss Microimaging, Thornwood, NY).

For chondrogenic studies, cells were seeded into 24-well plates at a density of 8 x 10^4 cells/5 µL drops to generate micromass cultures. After 2 h (Day 0), media were changed to StemPro Chondrogenic Basal Media (Gibco, Thermo Fisher Scientific, Waltham, MA), 10 % FBS, 1 % P/S (Control media) or StemPro Chondrogenic Differentiation Kit Media (Gibco, Thermo Fisher Scientific, Waltham, MA; Chondrogenic media). Media were changed every 72 h. After 24 days, cells were washed with PBS, fixed with 10 % neutral buffered formalin for 30 min, stained with 1 % Alcian Blue (in 1N HCl; Electron Microscopy Sciences, Hatfield, PA) for 30 min, washed three times with distilled water and air-dried. Brightfield images were acquired using an inverted Zeiss AxioObserver Z1 microscope equipped with an Axiocam ICC 1 color camera.

For osteogenic studies involving osteogenic gene expression, cells were seeded into 24-well plates at a density of 1.57 x 10^4 cells/cm^2 overnight. The following day (Day 0), media were changed to DMEM, 10 % FBS, 1 % P/S (Without BMP-2) or DMEM, 10 % FBS, 1 % P/S, 100 ng/mL BMP-2 (With BMP-2; Infuse Bone Graft, Medtronic, Sunnyvale, CA). Media were changed every 72 h. After 6 days, cells were dissociated with 0.25 % trypsin, the RNA harvested (Qiagen RNeasy Plus Mini kit, Qiagen, Germany) and reverse-transcribed into cDNA (Omniscript kit, Qiagen, Germany). Quantitative, real-time PCR was performed on the cDNA templates for 40 cycles using Taqman Gene Expression Mastermix (4369016, Applied Biosystems, Thermo Fisher Scientific, Waltham, MA) with *alkaline phosphatase* (*alp*, forward primer: CAAGAACAGAACCGATGTGG, reverse primer: TGTGTTTCGGTTTGAAGCTC and 5' FAM / 3' TAMRA probe: CGTCCAGCCTCGTGCCTCTG), *osteocalcin* (*ocn*, forward primer: GAGCTCAACCCTGACTGTGA, reverse primer: CTAGACTGGGCCGTAGAAGC and 5' FAM / 3' TAMRA probe: CCACATCGGCTTCCAGGAAGC), *osteoblast specific factor-1* (*osf1*, forward primer: GATCTGAACACGGCTCTGAA, reverse primer: TTGAGGTTTGGACTTGGTCA and 5' FAM / 3' TAMRA probe: AGCCTGAAGCGAGCCCTCCA) and *runt related transcription factor-2* (*runx2*, forward primer: GAGATCGGTCTCCTTCCAGA, reverse primer: TTAATAGCGTGCTGCCATTC and 5' FAM / 3' TAMRA probe: ATGCTACCGCCGTGCACCAC) primers. *18s ribosomal rna* (*18s*, forward primer: AGGTGAAATTCTTGGATCGG, reverse primer: GGTCGGAACTACGACGGTAT and 5' FAM / 3' TAMRA probe: TCGCTCTGGTCCGTCTTGCG) primer was used as a reference gene for normalization. These primers were designed using a consensus sequence of homologous genes for bovine, human, mouse and rat species. Quantitative, real-time PCR was performed on an Applied Biosystems HT7200 thermocycler (Applied Biosystems, Thermo Fisher Scientific, Waltham, MA). Data were analyzed using SDS 2.2.2 (Applied Biosystems, Thermo Fisher Scientific, Waltham, MA, http://www.thermofisher.com/).

For osteogenic studies involving ALP staining, cells were seeded into 24-well plates at a density of 1.57 x 10^4 cells/cm^2 overnight. The following day (Day 0), media were changed to DMEM, 10 % FBS, 1 % P/S (Without BMP-2) or DMEM, 10 % FBS, 1 % P/S, 100 ng/mL BMP-2 (With BMP-2). Media were changed every 48 h. After 6 days, cells were fixed for 1 min in 3.7 % formaldehyde. ALP activity

(Kit 86C, Sigma Aldrich, St. Louis, MO) was detected according to the manufacturer's instructions. Brightfield images were acquired using an inverted Zeiss AxioObserver Z1 microscope equipped with an Axiocam ICC 1 color camera as well as a Nikon digital SLR camera (Nikon Digital Camera D70, Nikon Corp., Japan). Where necessary, the average pixel intensity was determined using the image histogram tool in Adobe Photoshop (Adobe Systems, San Jose, CA, http://www.adobe.com) as previously described (2, 3).

For osteogenic studies involving Alizarin Red S staining, cells were seeded into 24-well plates at a density of 1.57 x 10^4 cells/cm^2 overnight. The following day (Day 0), media were changed to DMEM, 10 % FBS, 1 % P/S, 50 µg/mL ascorbic acid (Sigma Aldrich, St. Louis, MO), 10 mM β-glycerophosphate (Sigma Aldrich, St. Louis, MO; Without BMP-2 and dexamethasone) or DMEM, 10 % FBS, 1 % P/S, 50 µg/mL ascorbic acid, 10 mM β-glycerophosphate, 100 ng/mL BMP-2 and 100 nM dexamethasone (Sigma Aldrich, St. Louis, MO; With BMP-2 and dexamethasone). Media were changed every 72 h. After 24 days, cells were fixed with 10 % neutral buffered formalin for 30 min, washed with distilled water, stained with 2 % Alizarin Red S stain (Electron Microscopy Sciences, Hatfield, PA) for 45 min, washed three times with distilled water and air-dried. Brightfield images were acquired using an inverted Zeiss AxioObserver Z1 microscope equipped with an Axiocam ICC 1 color camera as well as a Nikon digital SLR camera. To quantify Alizarin Red S staining, 1 mL of extraction solvent (8 % acetic acid, 20 % methanol in water) was added to each well for 45 min. Standards were constructed using 0, 0.1, 0.5, 1, 2, 5, 10, 50, 100, 200, 500, 700 µg/mL Alizarin Red S stain. Absorbance of standards and samples were read at 405 nm using a Tecan Infinite F50 spectrometer (Tecan Trading AG, Switzerland).

Construction of FD RM cell and hMSC cDNA libraries for RNA-seq. RNA-seq studies were performed for FD RM cells (Isolate 2) and hMSCs (Isolate 24268) under proliferation and mineralization conditions to identify proliferation and mineralization genes, respectively.

For proliferation studies, both FD RM cells and hMSCs were seeded at a density of 1.26 x 10^4 cells/cm^2 overnight and media conditions included 1) DMEM, 0 % FBS, 1 % P/S (0 % serum) and 2) DMEM, 10 % FBS, 1 % P/S (10 % serum). In parallel, another set of cells was seeded at similar densities in 48-well plates to monitor cell growth daily using an automated cell counter (For 6 days). Media were changed every 48 h. After 2.5 days (when cells were observed to be in the exponential phase of growth), RNA was harvested.

For mineralization studies, both FD RM cells and hMSCs were seeded at a density of 1.26 x 10^4 cells/cm^2 overnight and media conditions included 1) DMEM, 10 % FBS, 1 % P/S, 50 µg/mL ascorbic acid and 10 mM β-glycerophosphate (Control media; Without BMP-2 and dexamethasone) and 2) DMEM, 10 % FBS, 1 % P/S, 50 µg/mL ascorbic acid, 10 mM β-glycerophosphate, 100 ng/mL BMP-2 and 100 nM dexamethasone (Osteogenic media; With BMP-2 and dexamethasone). In parallel, another set of cells was seeded at similar densities in 24-well plates to monitor cell mineralization using Alizarin Red S staining at 24 days. Media were changed every 72 h. After 24 days, RNA was harvested.

For each condition, two replicate RNA samples were isolated. Cells were dissociated with 0.25 % trypsin, the RNA harvested (Qiagen RNeasy Plus Mini kit, Qiagen, Germany) and reverse-transcribed into cDNA (Ovation RNA-seq System V2 kit, NuGEN, San Carlos, CA) for RNA-seq library construction. First, the remainder of the cDNA was sheared (S2 focused-ultrasonicator, Covaris, Woburn, MA). Following this, end repair of the fragmented cDNA, dA-tailing of the end-repaired cDNA, adaptor ligation of dA-tailed cDNA (using custom primers), and PCR enrichment of adaptor-ligated cDNA were performed for 6 cycles to prepare the cDNA library for RNA-Seq (NEBNext DNA Library Prep Master Mix Set for Illumina, New England Biolabs, Ipswich, MA). Human and FD proliferation samples were sequenced to approximately 87,276,798 reads per library while human and FD mineralization samples were sequenced to approximately 74,904,447 reads per library (**Supplementary Table 1** and **2**).

RNA-seq and bioinformatics analysis. After cDNA library preparation, samples were sequenced using 100 base-pair, paired-end RNA-seq technology (HiSeq 2000, Illumina, San Diego, CA) and data were analyzed using several bioinformatics software (4, 5). The raw sequencing data were concatenated as necessary and the adaptor sequences were removed from the reads to prepare them for analysis. In order to analyze the data, all reads were aligned to the appropriate genome using Spliced Transcripts Alignment to a Reference (STAR; Version 2.3.0, https://code.google.com/p/rna-star/) software (4). Both FD RM proliferation and mineralization reads were aligned to the bovine (*Bos taurus*) genome (bosTau7 from UCSC Genome Browser, indexed using STAR), as *Bos taurus* is the closest relative to the fallow deer whose genome has been sequenced (at the time of analysis). Since the human genome is readily available, both hMSC proliferation and mineralization reads were aligned to the human (*Homo sapiens*) genome (pre-indexed hg19 released by STAR authors). After converting the STAR output to the appropriate file type and sorting the files using SAMtools (Version 0.1.19 http://www.htslib.org/), the Cufflinks package (Version 2.1.1.1, https://github.com/cole-trapnell-lab/cufflinks) was used to assemble the gene transcripts and to determine differentially-expressed genes between control and treatment conditions (5). Initial attempts at running the Cufflinks package on both FD RM cell and hMSC datasets resulted in errors. These errors originated from Cufflinks' treatment of the soft-clipped regions of aligned reads from the STAR output for some scaffolds (Alexander Dobin, personal communications). When these error-causing soft-clipped regions were removed from both FD and human datasets, the Cufflinks package ran with no errors. The output from Cufflinks was subsequently processed in R-Studio using the cummerbund package (http://compbio.mit.edu/cummeRbund/) to visualize RNA-seq data as well as data quality. Differentially-expressed genes between control and treatment groups were identified based on cut-off values for probability ($p \leq 0.05$) and false discovery rate ($q \leq 0.05$). Microsoft Excel (Microsoft Corp., Redmond, WA; http://www.microsoftstore.com/), Ingenuity Pathway Analysis (Qiagen, Germany; http://www.ingenuity.com/products/ipa) and Gene Ontology Enrichment Analysis (http://geneontology.org/page/go-enrichment-analysis) were used to compare and analyze differentially-expressed genes in FD RM cell and hMSC datasets. Genes-of-interest for subsequent cloning were identified based on two arbitrary criteria – 1) Differentially-expressed genes that exhibited more than 5-fold upregulation in control versus treatment conditions and 2) Genes that were uniquely-expressed in the FD RM cell dataset (i.e. not differentially-expressed in the hMSC dataset).

Cloning of FD genes. Uniquely-expressed, FD genes were PCR-cloned, ligated into a plasmid, transformed into bacteria and purified for subsequent overexpression studies in mammalian cells.

To perform PCR-based cloning, primers were designed to isolate the gene-of-interest from the cDNA of FD RM cells based on the sequences of homologous genes in the bovine genome (National Center for Biotechnology Information, http://www.ncbi.nlm.nih.gov/). These primers included complementary regions 15 – 20 base pairs into the 5' and 3' ends of the bovine genes, and contained enzyme restriction sites necessary for eventual insertion into the pVitro2-MCS-Blast plasmid (InvivoGen, San Diego, CA), which contains 2 multiple cloning sites (MCS), a blasticidin resistance gene as well as necessary elements for bacterial and mammalian expression. Each gene was checked to ensure that the selected enzyme restriction sites were not present within the coding sequence of the published mRNA sequence. PCR (Platinum Blue PCR SuperMix, Invitrogen, Thermo Fisher Scientific, Waltham, MA) was performed according to the manufacturer's instructions. PCR products were purified (Wizard SV Gel and PCR Clean-up System, Promega, Sunnyvale, CA) and successful cloning was confirmed via gel electrophoresis and DNA sequencing (ElimBio, Hayward, CA).

To construct the plasmid containing the gene-of-interest, purified PCR products were digested using the appropriate restriction enzymes (New England Biolabs, Ipswich, MA), ligated into pVitro2-MCS-Blast plasmid according to the manufacturer's instructions (NEB Quick Ligation Kit, New

England Biolabs, Ipswich, MA). Successful construction of the plasmid containing the gene-of-interest was confirmed by DNA sequencing.

To obtain large quantities of DNA for subsequent overexpression studies, plasmids were transformed into chemically competent DH5α *E. coli* (One Shot MAX Efficiency DH5α-T1 Competent Cells, Invitrogen, Thermo Fisher Scientific, Waltham, MA) and purified. Bacterial cultures were amplified on LB-agar-blasticidin (Invivogen, San Diego, CA) plates in a 5 % CO_2 incubator at 37 °C overnight. Successful colonies were grown in TB-blasticidin liquid media at 37 °C, shaking at 225 – 250 rpm overnight, before isolating the amplified plasmid DNA (Purelink Quick Plasmid Miniprep Kit or Purelink HiPure Plasmid Midiprep Kit, Invitrogen, Thermo Fisher Scientific, Waltham, MA). Several plasmids including pVitro2-MCS-Blast (empty plasmid) as well as pVitro2-*mRuby2*-Blast (6), a plasmid encoding the fluorescent protein mRuby2 (7), were similarly obtained to serve as appropriate transfection controls.

Stable transfection of FD genes into C3H10T1/2 cells. C3H10T1/2 cells were stably-transfected with plasmid(s) containing the gene-of-interest for overexpression studies. C3H10T1/2 cells were seeded into 6-well plates at a density of 0.67 x 10^4 cells/cm^2 (60 – 70 % confluency) overnight. The following day, media were changed to DMEM, 10 % FBS, 1 % P/S and cells were transfected with 2 – 3 µg plasmid according to the manufacturer's instructions (Polyplus, France) for 24 h. After 24 h transfection, blasticidin-resistant cells were selected in DMEM, 10 % FBS, 1 % P/S and 3 µg/mL blasticidin (Invitrogen, Thermo Fisher Scientific, Waltham, MA) for 10 days. Media were changed every 48 h. Post-selection, blasticidin-resistant cells were maintained in DMEM, 10 % FBS, 1 % P/S. Untransfected cells and cells transfected with pVitro2-MCS-Blast or pVitro2-*mRuby2*-Blast were used as transfection controls.

Confirmation of gene overexpression using non-competitive, semi-quantitative PCR. To confirm gene overexpression, non-competitive, semi-quantitative PCR was employed. RNA was harvested from untransfected cells, cells stably transfected with the empty pVitro2-MCS-Blast plasmid and cells stably transfected with the pVitro2-MCS-Blast plasmids containing the gene-of-interest (Qiagen RNeasy Plus Mini kit, Qiagen, Germany) under appropriate culture conditions. The RNA samples were reverse-transcribed into cDNA according to the manufacturer's instructions. PCR was performed on the cDNA templates for 22 – 35 cycles with primers used to originally clone the gene-of-interest as well as Bovine *gapdh* as a reference gene for normalization. PCR-amplified DNA was separated by on a 1.0 % agarose gel (Sigma Aldrich, St. Louis, MO) at 80V for 1 – 1.5 h (Bio-Rad Laboratories Inc., Hercules, CA). Images of gel electrophoresis samples were analyzed using the image histogram tool in Adobe Photoshop as previously described (2, 3).

Proliferation capability of identified FD gene(s). The function of FD gene(s)-of-interest in cell proliferation was measured in stably-transfected C3H10T1/2 cells using cell counting studies and in FD RM cells using gene knockdown studies.

For cell counting studies, cells were seeded into 48-well plates at a density of 0.26 x 10^4 cells/cm^2 overnight (Day 0) in DMEM, 10 % FBS, 1 % P/S. Media were changed every 48 h. Cells were counted daily using an automated cell counter for 5 days. Cell doubling times were calculated using R-studio (R Studio, Boston, MA, http://www.rstudio.com) by visually determining the exponential phase of growth, and plotting the log of the cell counts against time to determine the slope (ln(2)/slope = doubling time) for each sample.

For gene knockdown studies, cells were seeded into 48-well plates at a density of 0.26 x 10^4 cells/cm^2 overnight in DMEM, 10 % FBS, 1 % P/S. The following day (Day 0), cells were transfected with 30 nM *uhrf1* siRNA A and E (Custom-designed based on bovine *uhrf1* sequence, Santa Cruz Biotechnology Inc., Dallas, TX) according to the manufacturer's instructions (Polyplus, France) for 72 h. No media change was performed. Confirmation of siRNA-mediated *uhrf1*

knockdown was determined using non-competitive, semi-quantitative PCR and gel electrophoresis. At 0 and 3 days, cells were counted using an automated cell counter.

Osteogenic differentiation capability of identified FD gene(s). The function of FD gene(s)-of-interest in osteogenic differentiation was measured in stably-transfected C3H10T1/2 cells using osteogenic gene expression, ALP staining, and Alizarin Red S staining.

For osteogenic gene expression, cells were seeded into 24-well plates at a density of 1.57 x 10^4 cells/cm^2 overnight. The following day (Day 0), media were changed to DMEM, 10 % FBS, 1 % P/S (Without BMP-2) or DMEM, 10 % FBS, 1 % P/S, 100 ng/mL BMP-2 (With BMP-2). Media were changed every 72 h. At appropriate timepoints (4 h or 12 days), cells were dissociated with 0.25 % trypsin, the RNA harvested and reverse-transcribed into cDNA. Quantitative, real-time PCR was performed on the cDNA templates for 40 cycles using Taqman Gene Expression Mastermix with *alkaline phosphatase* (*alp*; Mm00475834_m1, Applied Biosystems, Thermo Fisher Scientific, Waltham, MA), *osteocalcin* (*ocn*; Mm03413826_mH, Applied Biosystems, Thermo Fisher Scientific, Waltham, MA) and *runt related transcription factor 2* (*runx2*; Mm00501584_m1, Applied Biosystems, Thermo Fisher Scientific, Waltham, MA) primers. *18s ribosomal rna* (*18s*; Mm03928990_g1, Applied Biosystems, Thermo Fisher Scientific, Waltham, MA) was used as a reference gene for normalization. Quantitative, real-time PCR was performed on an Applied Biosystems HT7200 thermocycler. Data were analyzed using SDS 2.2.2 (Applied Biosystems, Thermo Fisher Scientific, Waltham, MA, http://www.thermofisher.com/).

For ALP staining, cells were seeded into 24-well plates at a density of 1.57 x 10^4 cells/cm^2 overnight. The following day (Day 0), media were changed to DMEM, 10 % FBS, 1 % P/S (Without BMP-2) or DMEM, 10 % FBS, 1 % P/S, 100 ng/mL BMP-2 (With BMP-2). Media were changed every 48 h. After 4 – 12 days, cells were fixed for 1 min in 3.7 % formaldehyde. ALP activity was detected according to the manufacturer's instructions. Brightfield images were acquired using an inverted Zeiss AxioObserver Z1 microscope equipped with an Axiocam ICC 1 color camera. Where necessary, the average pixel intensity was determined using the image histogram tool in Adobe Photoshop as previously described (2, 3).

For Alizarin Red S staining, cells were seeded into 24-well plates at a density of 1.57 x 10^4 cells/cm^2 overnight. The following day (Day 0), media were changed to DMEM, 10 % FBS, 1 % P/S, 50 µg/mL ascorbic acid, 10 mM β-glycerophosphate (Without BMP-2 and dexamethasone) or DMEM, 10 % FBS, 1 % P/S, 50 µg/mL ascorbic acid, 10 mM β-glycerophosphate, 100 ng/mL BMP-2 and 100 nM dexamethasone (With BMP-2 and dexamethasone). Media were changed every 72 h. After 24 days, cells were fixed with 10 % neutral buffered formalin for 30 min, washed with distilled water, stained with 2 % Alizarin Red S stain for 45 min, washed three times with distilled water and air-dried. Brightfield images were acquired using an inverted Zeiss AxioObserver Z1 microscope equipped with an Axiocam ICC 1 color camera. To quantify Alizarin Red S staining, 1 mL of extraction solvent (8 % acetic acid, 20 % methanol in water) was added to each well for 45 min. Standards were constructed using 0, 0.1, 0.5, 1, 2, 5, 10, 50, 100, 200, 500, 700 µg/mL Alizarin Red S stain. Absorbance of standards and samples were read at 405 nm using a Tecan Infinite F50 spectrometer.

Immunofluorescence staining of FD RM cells. To ascertain *in vitro* physiological relevance of *in vitro* comparative RNA-seq results, FD RM cells were subjected to immunofluorescence staining under proliferation and mineralization conditions. For proliferation studies, FD RM cells were seeded at a density of 1.26 x 10^4 cells/cm^2 overnight and media conditions included 1) DMEM, 0 % FBS, 1 % P/S (No growth factor control; Serum-free media), 2) Serum-free media supplemented with 100 ng/mL insulin-like growth factor-1 (IGF-1; Peprotech, Rocky Hill, NJ), 3) Serum-free media supplemented with 50 ng/mL FGF-2, 4) DMEM, 10 % FBS, 1 % P/S (No growth factor control; Serum-containing media), 5) Serum-containing media supplemented with 100 ng/mL IGF-1 and 6) Serum-containing media supplemented with 50 ng/mL FGF-2. Immunofluorescence staining was performed after 2 days. For mineralization studies, FD RM cells were seeded at a density of 1.26 x

10^4 cells/cm^2 overnight and media conditions included 1) DMEM, 10 % FBS, 1 % P/S, 50 μg/mL ascorbic acid and 10 mM β-glycerophosphate (Control; Without BMP-2 and dexamethasone media) and 2) DMEM, 10 % FBS, 1 % P/S, 50 μg/mL ascorbic acid, 10 mM β-glycerophosphate, 100 ng/mL BMP-2 and 100 nM dexamethasone (Treatment; With BMP-2 and dexamethasone media). Media were changed every 72 h and immunofluorescence staining was performed after 24 days.

For immunofluorescence staining, cells were fixed in 4 % paraformaldehyde, washed 3 times in PBS, permeabilized with 0.2 % Triton X-100 for 10 min and washed 3 times in PBS. Following this, antibody staining was performed. Cells were incubated in 10 % donkey serum for 20 min followed by incubation with 10 μg/mL rabbit anti-UHRF1 (Sc98704, Santa Cruz Biotechnology Inc., Dallas, TX) or 1 μg/mL mouse anti-S100A10 (Ab89438, Abcam Inc., Cambridge, MA) primary antibody overnight at 4 °C. The following day, cells were washed 3 times in wash buffer (5 min each), incubated in 15 μg/mL donkey anti-rabbit Alexa 647 (711-605-152, Jackson Immunoresearch, West Gove, PA) or 15 μg/mL donkey anti-mouse Alexa 647 (715-605-150, Jackson Immunoresearch, West Gove, PA) secondary antibody for 1 h at 25 °C and washed 5 times in wash buffer (5 min each). Fluorescence images were acquired using an inverted Zeiss AxioObserver Z1 microscope equipped with an X-Cite® Series 120Q metal halide lamp, appropriate filters and an AxioCam MRm camera. Where necessary, the average pixel intensity was determined using the image histogram tool in Adobe Photoshop as previously described (2, 3).

Histological and immunofluorescence staining of antler tissue. To examine antler tissue regeneration and ascertain *in vivo* physiological relevance of *in vitro* comparative RNA-seq results, deer antler tissue were harvested from an independent fallow deer herd at another local deer ranch (Walking Beam Ranch, Santa Paula, CA) in accordance with the guidelines established by Stanford University's Administrative Panel on Laboratory Animal Care and subjected to histological and immunofluorescence staining. These deer were approximately 2 – 3 years old and antler tissues were harvested during early stages of antler regeneration (4 – 7 inches in height) as described previously. Tissue samples were fixed in 10 % formalin and stored in 70 % ethanol. Tissue samples were subjected to histological processing via a graded ethanol dehydration series at 4 °C (70 % ethanol overnight, 85 % ethanol overnight, 95 % ethanol overnight and 100 % ethanol overnight) followed by xylene infiltration at 25 °C (50 % xylene in ethanol for 1 h, 50 % xylene in ethanol overnight, 100 % xylene for 1 h and 100 % xylene overnight) and then paraffin infiltration at 60 °C (50 % paraffin in xylene for 2 h, 100 % paraffin for 2 h, 3 washes of 100 % paraffin for 20 min each, 100 % paraffin overnight and 100 % paraffin for 20 min). Subsequently, tissue samples were embedded in paraffin blocks and sectioned at 4 – 8 μm intervals using a Leica rotary microtome (RM 2255, Leica Biosystems Inc., Buffalo Grove, IL). Prior to staining, tissue sections were deparaffinized (3 washes of 100 % xylene for 3 min each) and rehydrated (50 % ethanol in xylene for 3 min, 2 washes of 100 % ethanol for 3 min each, 2 washes of 95 % ethanol for 3 min each, 2 washes of 70 % ethanol for 3 min each and PBS for 3 min).

For histological staining, tissue sections were stained with Alcian Blue or Alizarin Red S. To stain for cartilage, tissue samples were incubated with 1 % Alcian Blue (in 1N HCl) for 30 min, washed three times with distilled water, counter-stained with Neutral Red for 10 min, washed three times with distilled water, dehydrated in ethanol and mounted. Brightfield images were acquired using an inverted Zeiss AxioObserver Z1 microscope equipped with an Axiocam ICC 1 color camera. To stain for mineralized bone, thick (1 – 3 mm) tissue samples were incubated with 2 % Alizarin Red S for 30 min and washed five times with distilled water. Thick tissue samples were imaged using a Nikon digital SLR camera.

For immunofluorescence staining, antigen retrieval was performed using antigen retrieval buffer solution (IHC World LLC, Woodstock, MD) at 80 – 90 °C for 30 – 60 min prior to antibody incubation. Following this, antibody staining was performed. Tissue sections were blocked in 10 % donkey serum for 1 h followed by incubation with 10 μg/mL rabbit anti-UHRF1 (Sc98704, Santa Cruz Biotechnology

Inc., Dallas, TX) or 1 µg/mL mouse anti-S100A10 (Ab89438, Abcam Inc., Cambridge, MA) primary antibody overnight at 4 °C. The following day, tissue sections were washed 3 times with wash buffer (5 min each), incubated with 15 µg/mL donkey anti-rabbit Alexa 647 (711-605-152, Jackson Immunoresearch, West Gove, PA) or 15 µg/mL donkey anti-mouse Alexa 647 (715-605-150, Jackson Immunoresearch, West Gove, PA) secondary antibody for 1 h at 25 °C and washed 5 times with wash buffer (5 min each). Fluorescence images were acquired using an inverted Zeiss AxioObserver Z1 microscope equipped with an X-Cite® Series 120Q metal halide lamp, appropriate filters and an AxioCam MRm camera.

Statistical analysis. Statistical analyses involving RNA-seq were performed by Cufflinks and R-Studio (5). Statistical significance for differentially-expressed genes was established at $p \leq 0.05$ and $q \leq 0.05$. Statistical analyses not involving RNA-seq were performed using IBM SPSS Statistics for Windows 22 (IBM Corp., North Castle, NY, http://www.ibm.com). These experiments were performed with at least 3 replicates per condition. Sample sizes were estimated to detect a group mean difference of 50 % ± 1 to 2 standard deviations with a power (1 - β) of 0.8 and α = 0.05 (http://powerandsamplesize.com/Calculators/Compare-k-Means/1-Way-ANOVA-Pairwise).
Quantitative data was presented as means ± standard error of mean (mean ± SEM) where appropriate. Relative fold changes for PCR data were log transformed in order to make the data distribution more symmetrical since gene expression data are often log normally distributed (8). To determine whether data were normally-distributed and whether there was equality of variances among groups, *p* values were computed via the Shapiro-Wilk test and the Levene test, respectively. For two mean comparisons, *p* values were computed via the *t*-test. If there was equal variance between groups, *p* values were calculated using pooled variance. Otherwise, *p* values were calculated using separate variance. For more than two mean comparisons, *p* values were computed via Analysis of Variance (ANOVA). If majority of data (two-thirds or more) were normally-distributed or there was equal variance among groups, *p* values were calculated using ANOVA followed by Tukey's Honest Significant Difference *post-hoc* multiple comparison test. This approach was based on the robustness of ANOVA under conditions of non-normality and heterogeneity of variance (9, 10). Otherwise, *p* values were calculated using Welch's ANOVA followed by Games-Howell *post-hoc* multiple comparison test. This approach enables improved control of Type I errors and greater power under conditions of non-normality and heterogeneity of variance (11). Statistical significance was established at $p \leq 0.05$.

Supplementary Figures S1-S7

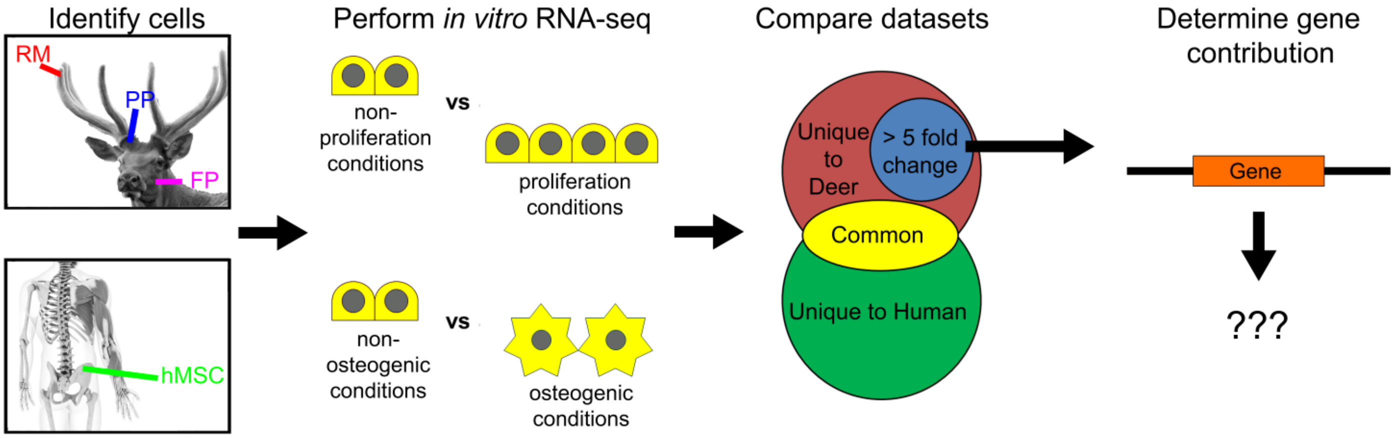

Supplementary Figure S1. Overview of *in vitro* comparative RNA-seq. The colored lines indicate sites where deer (FP, facial periosteum; PP, pedicle periosteum; RM, reserve mesenchyme) and human (hMSC, human mesenchymal stem cells) skeletal progenitor cells were harvested. Isolated cells were subjected to RNA-seq under proliferation and mineralization conditions independently and the resulting datasets were compared to identify genes that were highly-expressed (> 5-fold increase) and unique to deer. Subsequently, the physiological relevance and contribution of these genes to antler regeneration were determined.

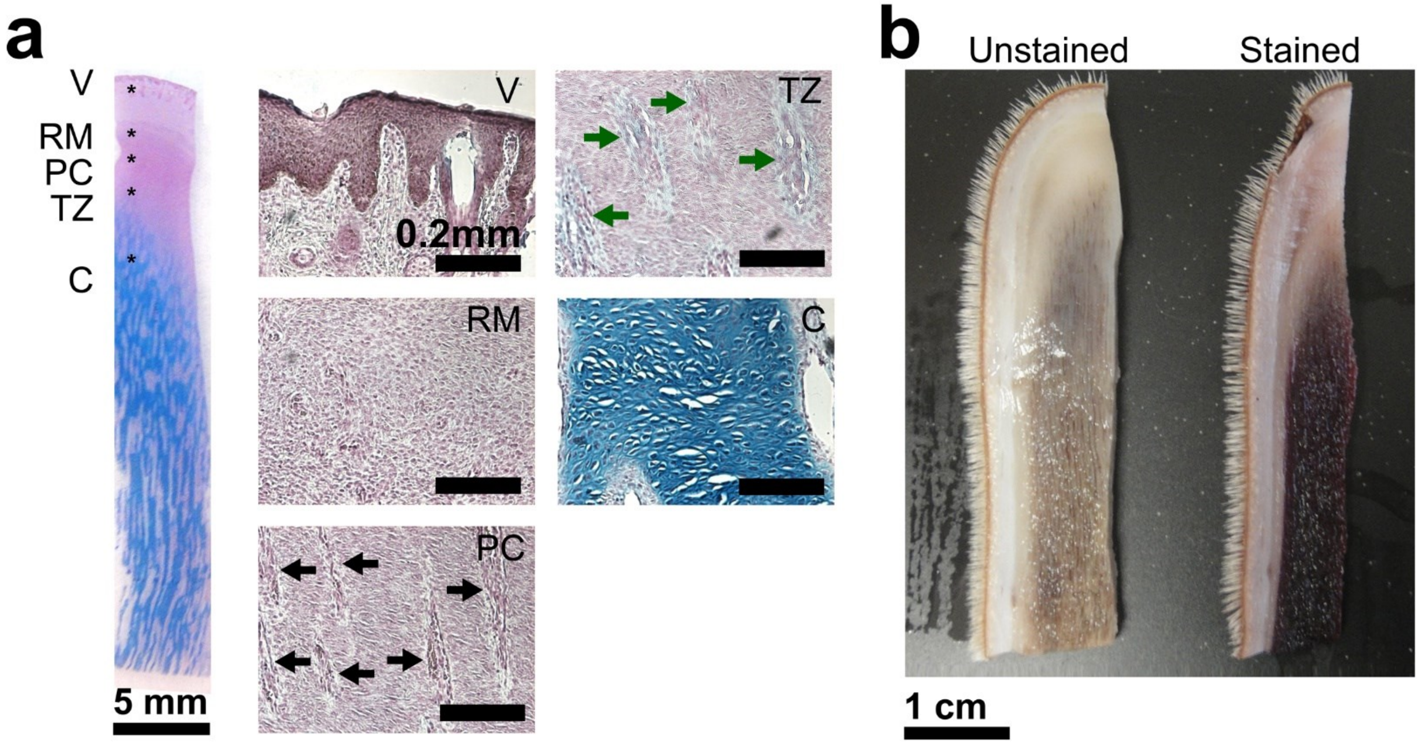

Supplementary Figure S2. Histological staining of regenerating deer antlers. (a) Alcian Blue staining of regenerating deer antler. The regenerating deer antler consists of velvet (V; skin), reserve mesenchyme (RM), pre-cartilage (PC), transitional zone (TZ) and cartilage (C) regions. PC, TZ and C regions indicate antler cells at different stages of endochondral ossification and are derived from RM tissue. Proximal and distal regions of deer antler stained positive and negative for Alcian Blue, respectively. Black arrows indicate discrete columns of pre-chondroblasts that stained negative for Alcian Blue. Green arrows indicate either discrete columns of chondroblasts or continuous columns of

cartilage cells that stained positive for Alcian Blue. Scale bars as indicated. **(b)** Alizarin Red S staining of regenerating deer antler. Proximal regions of deer antler stained positive for Alizarin Red S which are indicative of tissue calcification. Scale bars as indicated. Data were from n = 3 isolates.

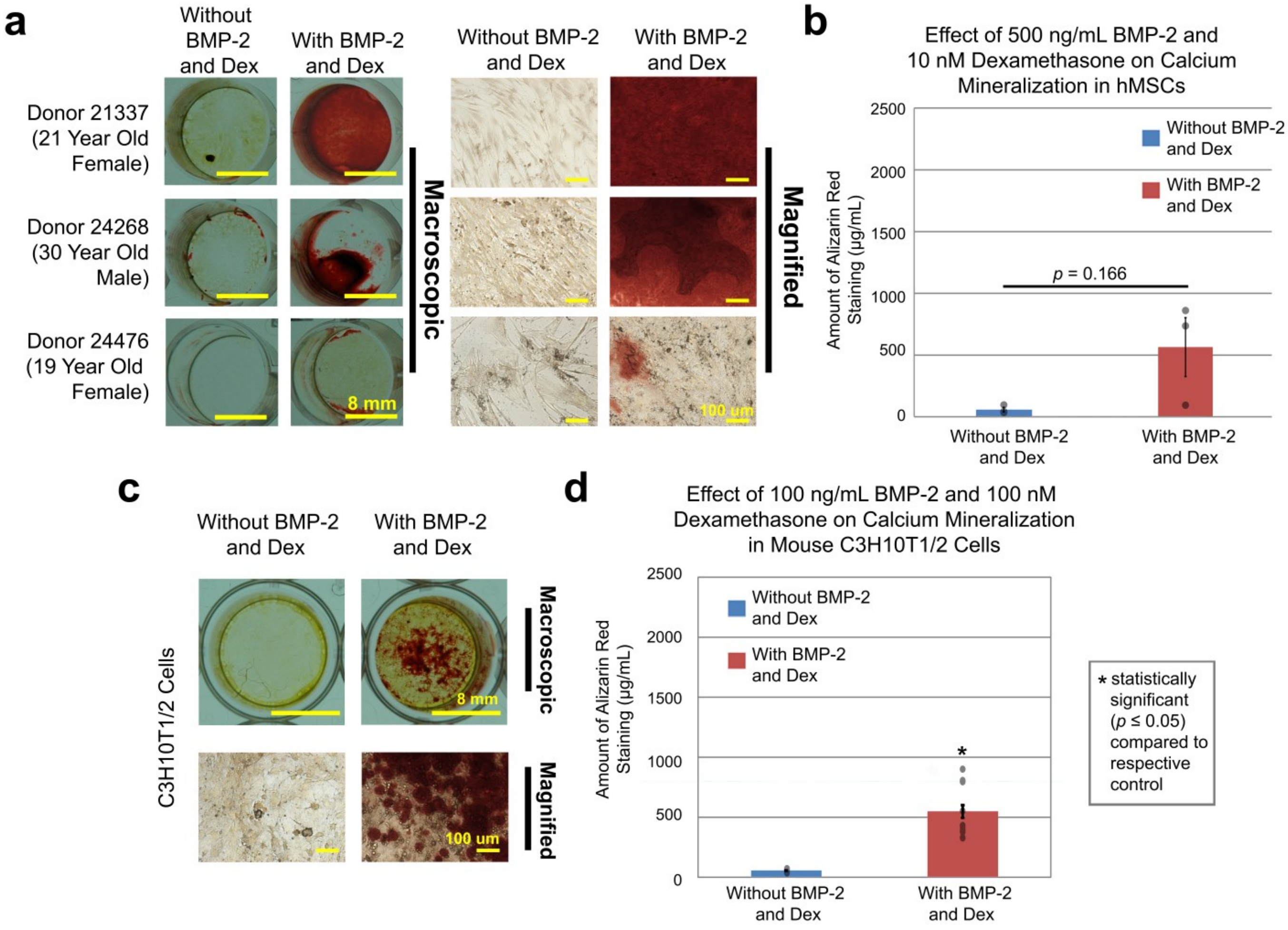

Supplementary Figure S3. Alizarin Red S staining of hMSCs and C3H10T1/2 cell under various mineralization conditions. (a) hMSC isolates 21337 and 24268 cultured with 500 ng/mL BMP-2 and 10 nM dexamethasone for 24 days exhibited increased Alizarin Red S staining relative to their respective control whereas hMSC isolate 24476 did not. **(b)** Quantification of Alizarin Red S staining in hMSCs. **(c)** C3H10T1/2 cells cultured with 100 ng/mL BMP-2 and 100 nM dexamethasone for 24 days exhibited increased Alizarin Red S staining relative to their respective control. **(d)** Quantification of Alizarin Red S staining in C3H10T1/2 cells. Scale bars as indicated. Data were from n = 3 isolates (3 independent experiments with 9 replicates per isolate) for hMSC studies and n = 2 independent experiments with 14 replicates per group for C3H10T1/2 cell studies. Grey circles indicate observed data points. Error bars indicate SEM. Statistical significance as indicated.

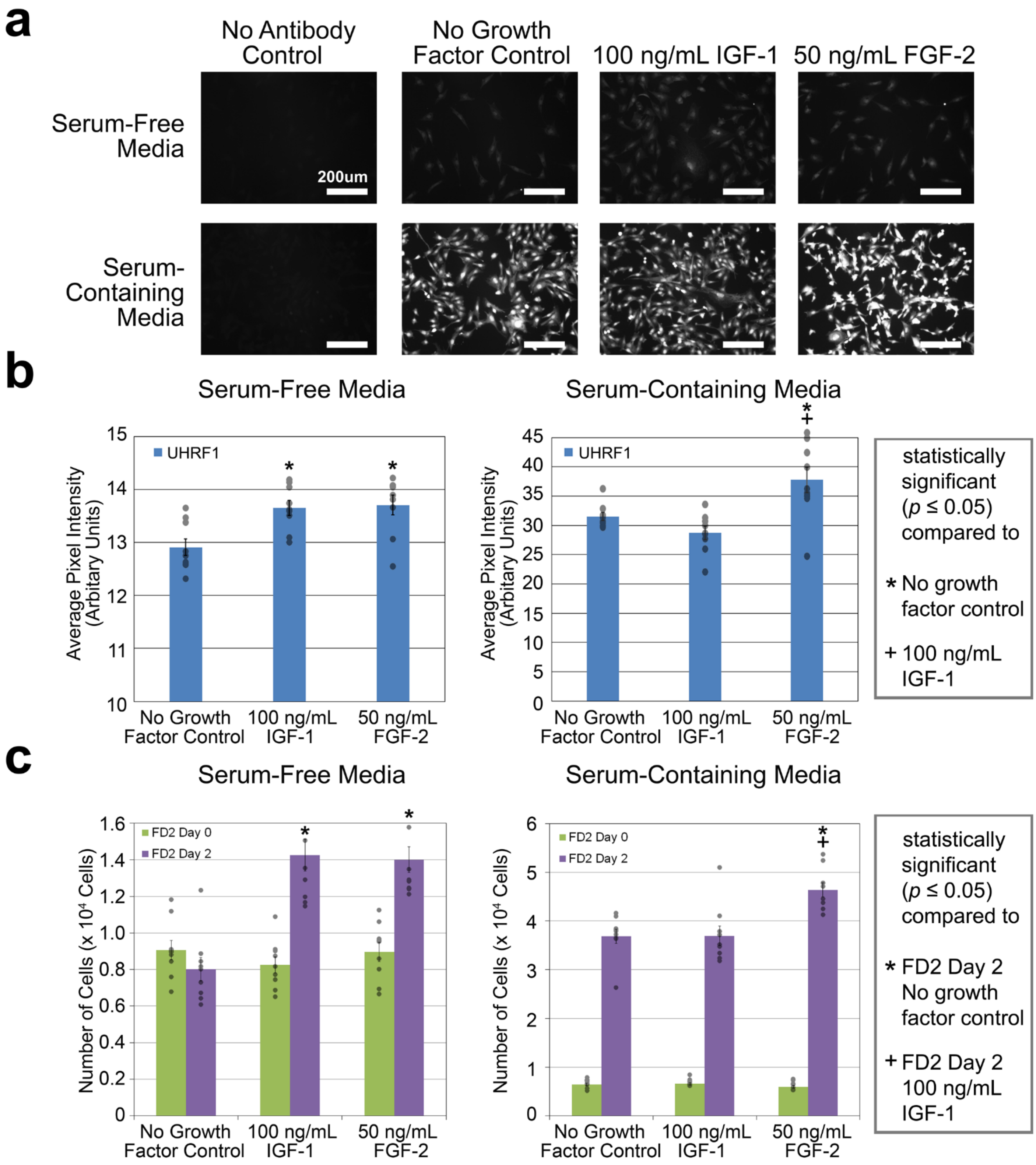

Supplementary Figure S4. IGF-1 and FGF-2 increased RM cell (Fallow Deer Isolate 2) proliferation and UHRF1 expression. (a) UHRF1 immunofluorescence staining in RM cells (Fallow Deer Isolate 2) cultured for 2 days under serum-free (0 % serum) and serum-containing (10 % serum) conditions in the absence or presence of 100 ng/mL IGF-1 and 50 ng/mL FGF-2. **(b)** Semi-quantification of UHRF1 expression in RM cells (Fallow Deer Isolate 2) cultured for 2 days under serum-free (0 % serum) and serum-containing (10 % serum) conditions in the absence or presence of 100 ng/mL IGF-1 and 50 ng/mL FGF-2. Under serum-free conditions, UHRF1 expression was

increased in RM cells in the presence of 100 ng/mL IGF-1 or 50 ng/mL FGF-2. Under serum conditions, UHRF1 expression was increased in RM cells in the presence of 50 ng/mL FGF-2. **(c).** Cell proliferation of RM cells (Fallow Deer Isolate 2) cultured for 2 days under serum-free (0 % serum) and serum-containing (10 % serum) conditions in the absence or presence of 100 ng/mL IGF-1 and 50 ng/mL FGF-2. Under serum-free conditions, RM cells exhibited increased proliferation in the presence of 100 ng/mL IGF-1 or 50 ng/mL FGF-2. Under serum conditions, RM cells exhibited increased proliferation in the presence of 50 ng/mL FGF-2. Scale bars as indicated. Data were from n = 3 independent experiments with 9 replicates per group. Grey circles indicate observed data points. Error bars indicate SEM. Statistical significance as indicated.

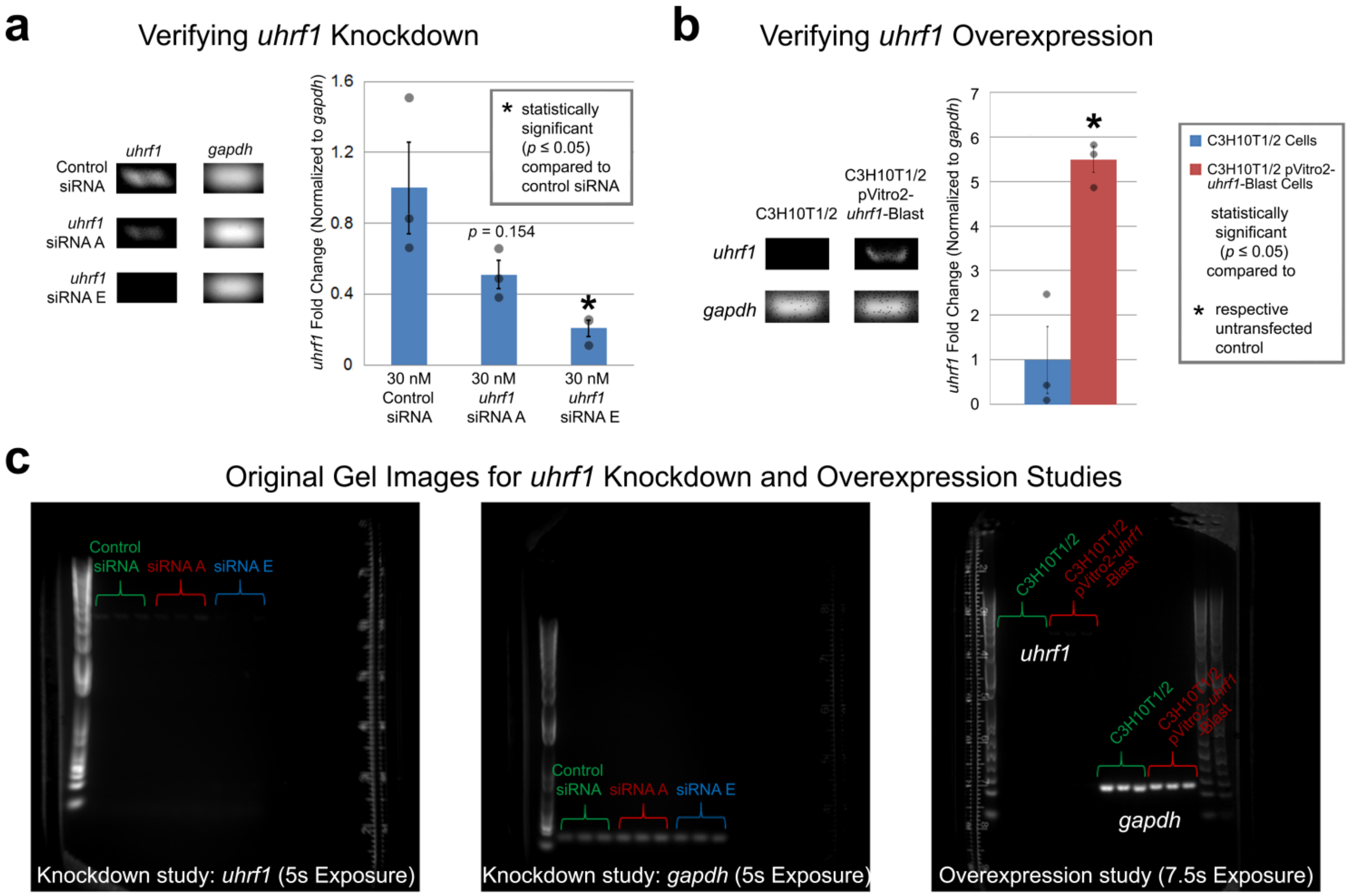

Supplementary Figure S5. siRNA-mediated gene knockdown of *uhrf1* in RM cells and overexpression of *uhrf1* in C3H10T1/2 cells. (a) RM cells cultured with 30 nM *uhrf1* siRNAs for 3 days exhibited decreased *uhrf1* expression relative to mock-transfected control. **(b)** C3H10T1/2 cells stably-transfected with *uhrf1* exhibited increased *uhrf1* gene expression relative to untransfected control. **(c)** Original images of DNA electrophoresis gels for *uhrf1* gene knockdown and *uhrf1* gene overexpression studies. Exposure time for capturing gel images are indicated in seconds. Data were from 1 independent experiment with 3 replicates per group. Grey circles indicate observed data points. Error bars indicate SEM. Statistical significance as indicated.

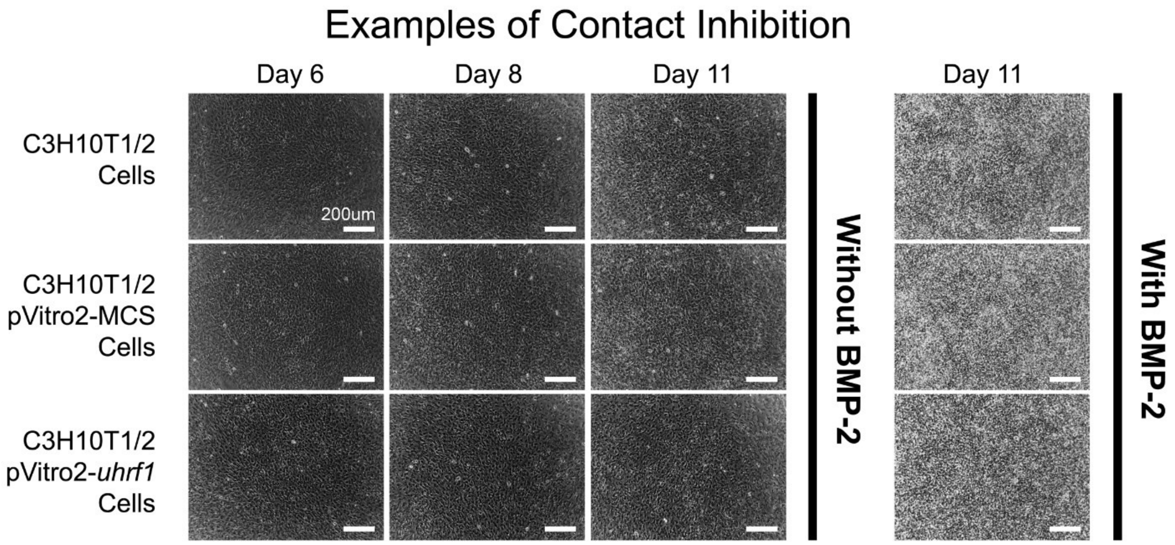

Supplementary Figure S6. Contact inhibition of C3H10T1/2 cells overexpressing FD *uhrf1*. C3H10T1/2 cells stably-transfected with *uhrf1* were contact inhibited. Representative images of confluent cultures at Days 6, 8 and 11 exhibit similar cell density in contrast to BMP-2-treated cells.

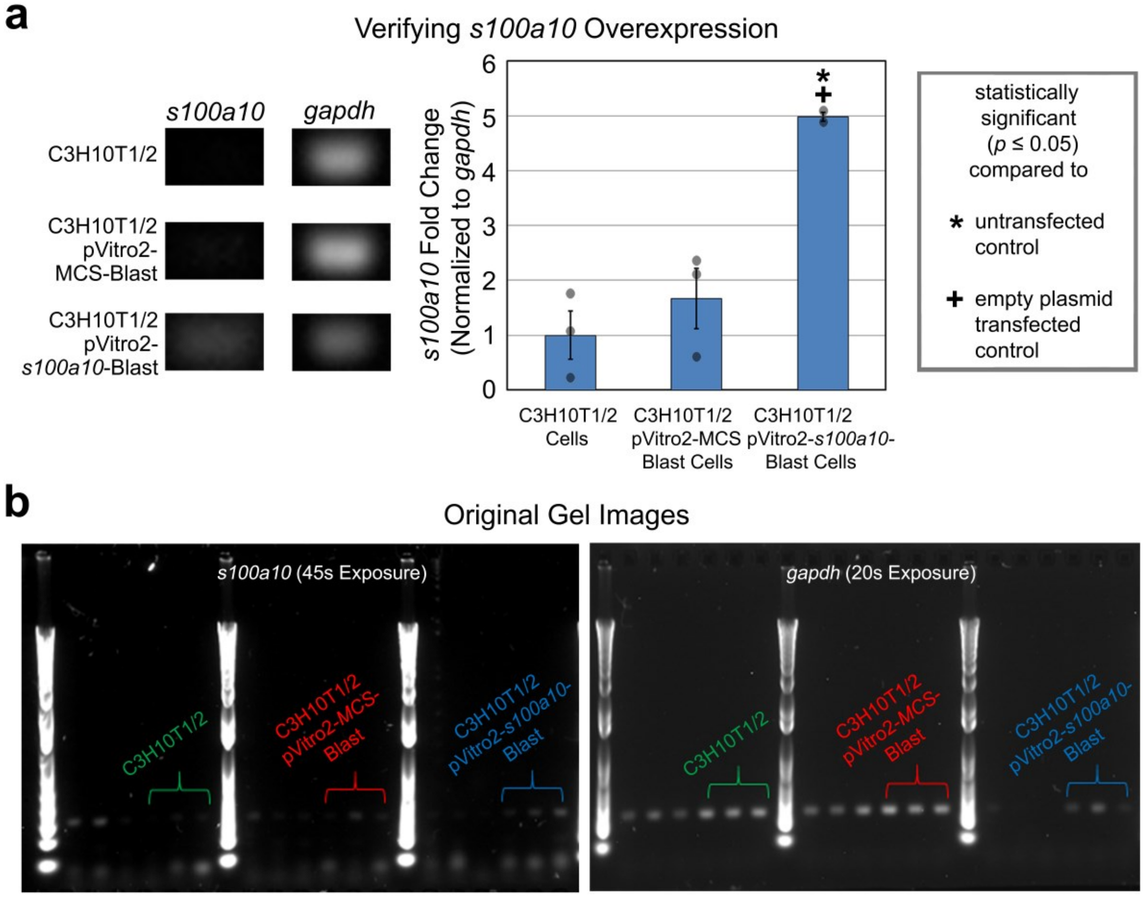

Supplementary Figure S7. Overexpression of FD *s100a10* in C3H10T1/2 cells. (a) C3H10T1/2 cells stably-transfected with *s100a10* exhibited increased *s100a10* gene expression relative to untransfected control and empty plasmid control. **(b)** Original images of DNA electrophoresis gels for *s100a10* gene overexpression studies. Exposure time for capturing gel images are indicated in seconds. Data were from n = 1 independent experiments with 3 replicates per group. Grey circles indicate observed data points. Error bars indicate SEM. Statistical significance as indicated.

Supplementary Tables

Supplementary Table S1. Data quality of FD RM cell (Isolate 2) RNA-Seq samples.

Sample	Yield (Mbases)	# Reads	% of raw clusters per lane	% of ≥ Q30 Bases (PF)	Mean Quality Score (PF)
0 Percent Serum 01	8,355	82,718,148	22.25	89.93	35.36
0 Percent Serum 02	8,549	84,639,852	22.77	89.88	35.33
10 Percent Serum 01	9,735	96,387,778	25.93	89.52	35.23
10 Percent Serum 02	8,552	84,671,322	22.78	89.59	35.2
0 ng/mL BMP-2 and 0 nM Dexamethasone 01	6,323	62,601,720	21.98	90.26	35.45
0 ng/mL BMP-2 and 0 nM Dexamethasone 02	7,915	78,363,548	27.52	91.15	35.77
100 ng/mL BMP-2 and 100 nM Dexamethasone 01	6,689	66,228,314	23.26	90.23	35.47
100 ng/mL BMP-2 and 100 nM Dexamethasone 02	6,729	66,627,578	23.4	90.58	35.54

Supplementary Table S2. Data quality of hMSC (Isolate 24268) RNA-seq samples.

Sample	Yield (Mbases)	# Reads	% of raw clusters per lane	% of ≥ Q30 Bases (PF)	Mean Quality Score (PF)
0 Percent Serum 01	7,755	76,781,962	21.27	88.47	34.79
0 Percent Serum 02	8,485	84,013,018	23.27	88.28	34.74
10 Percent Serum 01	9,018	89,286,210	24.73	89.17	35.04
10 Percent Serum 02	10,071	99,716,096	27.62	89.03	34.99
0 ng/mL BMP-2 and 0 nM Dexamethasone 01	7,320	72,477,298	21.48	89.61	35.16
0 ng/mL BMP-2 and 0 nM Dexamethasone 02	8,257	81,747,648	24.23	90.73	35.49
100 ng/mL BMP-2 and 100 nM Dexamethasone 01	8,762	86,750,048	25.72	90.17	35.34
100 ng/mL BMP-2 and 100 nM Dexamethasone 02	8,528	84,439,422	25.03	90.76	35.53

Supplementary references

1. Li C, Clark DE, Lord EA, Stanton JA, & Suttie JM (2002) Sampling Technique to Discriminate the Different Tissue Layers of Growing Antler Tips for Gene Discovery. *Anatomical Record* 268(2):125-130.
2. Ker ED*, et al.* (2011) Engineering Spatial Control of Multiple Differentiation Fates within a Stem Cell Population. *Biomaterials* 32(13):3413-3422.
3. Ker ED*, et al.* (2011) Bioprinting of Growth Factors onto Aligned Sub-Micron Fibrous Scaffolds for Simultaneous Control of Cell Differentiation and Alignment. *Biomaterials* 32(32):8097-8107.
4. Dobin A*, et al.* (2012) Star: Ultrafast Universal Rna-Seq Aligner. *Bioinformatics* 29:15-21.
5. Trapnell C*, et al.* (2012) Differential Gene and Transcript Expression Analysis of Rna-Seq Experiments with Tophat and Cufflinks. *Nature Protocols* 7(3):562-578.
6. Ker DFE, Sharma R, Wang ETH, & Yang YP (2015) Development of *mRuby2*-Transfected C3h10t1/2 Fibroblasts for Musculoskeletal Tissue Engineering. *PLoS One* 10(9):e0139054.
7. Lam AJ*, et al.* (2012) Improving Fret Dynamic Range with Bright Green and Red Fluorescent Proteins. *Nature Methods* 9(10):1005-1012.
8. Derveaux S, Vandesompele J, & Hellemans J (2010) How to Do Successful Gene Expression Analysis Using Real-Time Pcr. *Methods* 50(4):227-230.
9. Box GEP (1953) Non-Normality and Tests on Variances. *Biometrika* 40(3/4):318-335.
10. Box GEP (1954) Some Theorems on Quadratic Forms Applied in the Study of Analysis of Variance Problems, I. Effect of Inequality of Variance in the One-Way Classification. *Annals of Mathematical Statistics* 25(2):290-302.
11. Tomarken AJ & Serlin RC (1986) Comparison of Anova Alternatives under Variance Heterogeneity and Specific Noncentrality Structures. *Psychological Bulletin* 99(1):90-99.
